# Validating biomarkers and models for epigenetic inference of alcohol consumption from blood

**DOI:** 10.1186/s13148-021-01186-3

**Published:** 2021-10-26

**Authors:** Silvana C. E. Maas, Athina Vidaki, Alexander Teumer, Ricardo Costeira, Rory Wilson, Jenny van Dongen, Marian Beekman, Uwe Völker, Hans J. Grabe, Sonja Kunze, Karl-Heinz Ladwig, Joyce B. J. van Meurs, André G. Uitterlinden, Trudy Voortman, Dorret I. Boomsma, P. Eline Slagboom, Diana van Heemst, Carla J. H. van der Kallen, Leonard H. van den Berg, Melanie Waldenberger, Henry Völzke, Annette Peters, Jordana T. Bell, M. Arfan Ikram, Mohsen Ghanbari, Manfred Kayser

**Affiliations:** 1 Department of Epidemiology, Erasmus MC, University Medical Center Rotterdam, 3015 GD Rotterdam, The Netherlands; 2 Department of Genetic Identification, Erasmus MC, University Medical Center Rotterdam, 3015 GD Rotterdam, The Netherlands; 3 Institute for Community Medicine, University Medicine Greifswald, 17475 Greifswald, Germany; 4 DZHK (German Center for Cardiovascular Research), partner site Greifswald, 17475 Greifswald, Germany; 5 Department of Population Medicine and Lifestyle Diseases Prevention, Medical University of Bialystok, 15-269 Bialystok, Poland; 6 Department of Twin Research and Genetic Epidemiology, King’s College London, London, SE1 7EH UK; 7 Research Unit Molecular Epidemiology, Helmholtz Zentrum München, German Research Center for Environmental Health, 85764 Neuherberg, Germany; 8 Institute of Epidemiology, Helmholtz Zentrum München, German Research Center for Environmental Health, 85764 Neuherberg, Germany; 9 Netherlands Twin Register, Department of Biological Psychology, Vrije Universiteit, 1081 BT Amsterdam, The Netherlands; 10 Molecular Epidemiology, Department of Biomedical Data Sciences, Leiden University Medical Center, 2300 RC Leiden, The Netherlands; 11 Interfaculty Institute for Genetics and Functional Genomics, University Medicine Greifswald, Greifswald, Germany; 12 Department of Psychiatry and Psychotherapy, University Medicine Greifswald, Greifswald, Germany; 13 Department of Internal Medicine, Erasmus MC, University Medical Center Rotterdam, 3015 GD Rotterdam, The Netherlands; 14 Gerontology and Geriatrics, Department of Internal Medicine, Leiden University Medical Center, 2300 RC Leiden, The Netherlands; 15 Department of Internal Medicine, Maastricht University Medical Centre, 6229 EG Maastricht, The Netherlands; 16 Cardiovascular Research Institute Maastricht (CARIM), Maastricht University, 6229 EG Maastricht, The Netherlands; 17 Department of Neurology, Brain Center Rudolf Magnus, University Medical Center Utrecht, 3508 GA Utrecht, The Netherlands; 18 German Center for Cardiovascular Research (DZHK), Partner Site Munich Heart Alliance, 80802 Munich, Germany; 19 Institute for Medical Informatics, Biometrics, and Epidemiology, Ludwig-Maximilians-Universität (LMU) Munich, 81377 Munich, Germany

**Keywords:** Epigenetics, DNA methylation, Alcohol inference, Prediction, Inference, Blood

## Abstract

**Background:**

Information on long-term alcohol consumption is relevant for medical and public health research, disease therapy, and other areas. Recently, DNA methylation-based inference of alcohol consumption from blood was reported with high accuracy, but these results were based on employing the same dataset for model training and testing, which can lead to accuracy overestimation. Moreover, only subsets of alcohol consumption categories were used, which makes it impossible to extrapolate such models to the general population. By using data from eight population-based European cohorts (*N* = 4677), we internally and externally validated the previously reported biomarkers and models for epigenetic inference of alcohol consumption from blood and developed new models comprising all data from all categories.

**Results:**

By employing data from six European cohorts (*N* = 2883), we empirically tested the reproducibility of the previously suggested biomarkers and prediction models via ten-fold internal cross-validation. In contrast to previous findings, all seven models based on 144-CpGs yielded lower mean AUCs compared to the models with less CpGs. For instance, the 144-CpG heavy versus non-drinkers model gave an AUC of 0.78 ± 0.06, while the 5 and 23 CpG models achieved 0.83 ± 0.05, respectively. The transportability of the models was empirically tested via external validation in three independent European cohorts (*N* = 1794), revealing high AUC variance between datasets within models. For instance, the 144-CpG heavy versus non-drinkers model yielded AUCs ranging from 0.60 to 0.84 between datasets. The newly developed models that considered data from all categories showed low AUCs but gave low AUC variation in the external validation. For instance, the 144-CpG heavy and at-risk versus light and non-drinkers model achieved AUCs of 0.67 ± 0.02 in the internal cross-validation and 0.61–0.66 in the external validation datasets.

**Conclusions:**

The outcomes of our internal and external validation demonstrate that the previously reported prediction models suffer from both overfitting and accuracy overestimation. Our results show that the previously proposed biomarkers are not yet sufficient for accurate and robust inference of alcohol consumption from blood. Overall, our findings imply that DNA methylation prediction biomarkers and models need to be improved considerably before epigenetic inference of alcohol consumption from blood can be considered for practical applications.

**Supplementary Information:**

The online version contains supplementary material available at 10.1186/s13148-021-01186-3.

## Introduction

Alcohol consumption is a modifiable lifestyle factor associated with morbidity and mortality worldwide [1]. It was estimated to be the seventh-leading risk factor for disability-adjusted life-years (DALYs) and deaths in 2016, accounting for 5.2% (95% CI 4.4–6.0) of deaths globally [1]. Various diseases are caused or strongly influenced by excessive alcohol consumption, often in a dose-dependent manner, such as different forms of cancer, various liver diseases, cardiovascular disease, epilepsy, and unipolar depressive disorder [2].

Recent alcohol consumption is detectable by breathalyzers or direct measurement of the alcohol concentration in blood and urine; however, such measurements only provide information on few hours since the last alcohol consumption. For example, ethanol can be detected in urine within ten to twelve hours after the last drink, but not later [3]. Blood-based toxicological tests for alcohol consumption are also available, which are based on direct or indirect biomarkers. A direct biomarker is the result from ethanol metabolism or its reaction with other substances in the body, including ethyl glucuronide (EtG), ethyl sulfate (EtS), and phospholipid phosphatidylethanol (PEth). Indirect biomarkers are derived from cellular processes that undergo changes as a response to alcohol consumption, including carbohydrate-deficient transferrin (CDT), mean corpuscular volume (MCV), aspartate-aminotransferase (AST), alanine aminotransferase (ALT), and gamma-glutamyl transferase activity (GGT) [4, 5]. It is important to note that these direct and indirect biomarkers are specifically useful to determine the extreme categories, including excessive alcohol consumption or abstinence, and for recent alcohol consumption [5]. For example, CDT can distinguish excessive alcohol consumption of on average > 50–80 g ethanol per day over a period of 2 weeks [4]. In contrast, there are no reliable biomarkers available that can determine overall alcohol consumption habits like to distinguish heavy and at-risk drinkers from light and non-drinkers, or drinkers from non-drinkers and that are informative for alcohol consumption for longer periods of time. Therefore, due to the limited progress in previous alcohol biomarker research, information on long-term alcohol consumption is typically still collected using self-reports, although they are known to be unreliable [6]. Accurate and reliable biomarkers that reflect habitual alcohol consumption over months and years are needed to better diagnose and treat alcohol-related diseases and for objective exposure assessment in studies on alcohol consumption and health [7].

DNA methylation has been proposed as a biomarker for the detection of lifestyle factors in general [8] and several studies have already shown that alcohol consumption is associated with changes in DNA methylation levels in particular [9–12]. A few studies have also explored the possibility of epigenetic inference of alcohol consumption from blood [12–15]. A large benefit from epigenetic-based inference is the increasing availability of DNA methylation information in study participants, as DNA methylation is extensively studied for its association with diseases. The most extensive study investigating the epigenetic association and inference of alcohol consumption was done by Liu et al*.* [12]. In this study, an epigenome-wide association study (EWAS) meta-analysis on alcohol consumption was conducted in 9643 individuals of European ancestry from blood-derived DNA [12]. The authors identified 363 CpGs significantly associated (*P* < 1 × 10^−7^) with alcohol consumption levels used as a continuous variable (grams/day). A meta-analysis was performed for prediction marker discovery in a subset of 6926 participants of European ancestry, which identified 361 CpGs (*P* < 5 × 10^−6^). The study also reports impressively high prediction accuracies, expressed as area under the curve (AUC) estimates, for DNA methylation-based prediction models for categorical alcohol consumption based on sets of 5, 23, 78, or 144 CpG markers plus age, sex, and BMI. These models include pairwise combinations of four alcohol consumption categories with the highest AUC obtained for the models with the extreme categories. For instance, the reported 144-CpG model showed discrimination of heavy drinkers versus (vs.) non-drinkers with an AUC of 0.91–1.0 (an AUC of 1.0 means completely accurate inference) in the discovery dataset and all four replication cohorts as well as 0.86–1.0 for heavy drinkers vs. light drinkers [12]. The authors demonstrated increase in AUC with increased number of CpG predictors included in the models.

The high prediction accuracies reported by Liu et al*.* [12] were questioned based on methodological grounds by Hattab et al*.* [16]. Liu et al*.* were particularly criticized for not having used the coefficients from the discovery dataset to determine prediction accuracies in the replication datasets, but instead, they re-estimated these coefficients in each replication cohort using the same dataset for model training and testing. Hattab et al*.* [16] concluded that the prediction accuracies published by Liu et al*.* represent overestimates. However, Hattab et al*.* based their conclusions entirely on simulated data instead of empirical data. In a subsequent study, Yousefi et al*.* [17] found only half of the alcohol consumption variance explained by the DNA methylation markers in their independent data, compared to the explained variance values reported by Liu et al*.* [12]. In addition, Yousefi et al*.* [17] generated DNA methylation-derived scores using the coefficients made available by Liu et al*.*; based on these coefficients, they obtained much lower AUCs for the same models as reported by Liu et al. For instance, for adults at midlife, the reported AUCs were between 0.48 to 0.57 for distinguishing heavy drinkers from non-drinkers and AUCs between 0.55 to 0.57 for heavy drinkers vs. light drinkers. Although the Yousefi et al*.* study used empirical data, an important limitation of the study is their relatively small sample size*,* comprising only of 14 heavy drinkers, 67 at-risk drinkers, 748 light drinkers, and 54 non-drinkers.

Another source for the Liu et al*.* [12] AUCs putatively reflecting overestimations is their use of category subsets, and therewith participant subsets, in their prediction modeling approach. For instance, for estimating AUC for heavy drinkers vs. non-drinkers, Liu et al*.* only used data from heavy and non-drinkers thereby excluding the data from light drinkers and at-risk drinkers. Such use of partial data in prediction modeling is expected to result in overestimated prediction outcomes compared to a model that would include all available categories. Moreover, models that exclude participants based on their non-considered categories cannot be applied to the general populations where people with the excluded categories exist but can never be inferred correctly because their category was excluded from the model.

In the current study, we firstly aimed at replicating the association between alcohol consumption and the 363 CpGs previously identified by Liu et al*.* [12], using data from 2042 independent participants from five cohorts [18–22]. Then, by using a total of 4677 individuals from eight European cohorts [18–25], we aimed to thoroughly validate the DNA methylation biomarker sets and prediction models for the epigenetic inference of alcohol consumption from blood previously used by Liu et al. [12]. In addition, we trained and validated two new models including all alcohol consumption categories.

## Results

### Study populations and data sets

For replicating the association between alcohol consumption and the 363 CpGs previously reported by Liu et al*.* [12], we used data from 2042 individuals of five European cohorts as part of the Biobank-based Integrative Omics Study (BIOS) consortium [18–22, 26].

For prediction model building and internal validation, we employed a total dataset of 2883 Europeans, including the 2042 individuals from the BIOS consortium [26] together with 841 participants from The Cooperative Health Research in the Region of Augsburg (KORA) study (F4) [23]. Only participants with complete alcohol consumption data and DNA methylation data of all 144 predictive CpGs were included. Notably, there is no overlap between these data and those used by Liu et al. [12] in their prediction marker discovery EWAS. This makes our model building dataset completely independent from that of Liu et al. The KORA data included here were previously used by Liu et al*.* for prediction replication analysis; thus, its use for model building here provides no data dependency problem.

For external validation, we applied data from three European cohorts not applied for model training and internal validation: i.e., participants from the Rotterdam Study (sub-cohort RS-III-1) [18] (*N* = 648) not included in the BIOS consortium, from the Study of Health in Pomerania (SHIP)-Trend cohort (*N* = 433) [24], and two datasets from the TwinsUK Study, TwinsUK (*N* = 713) and TwinsUK2 (*N* = 442) [25]. The TwinsUK2 (*N* = 442) dataset comprises a subset of the TwinsUK (*N* = 713) participants but with re-processed DNA methylation dataset and a different alcohol consumption collection method (Additional file 1: Supplementary Methods). Of note, the TwinsUK and RS-III-1 data were previously used by Liu et al*.* [12] in their prediction marker discovery EWAS that identified the 361 associated CpGs (*P* < 5 × 10^–6^). Testing the inference ability of these 361 alcohol-associated CpGs by Liu et al. was solely conducted in the Framingham Heart Study data [27], which identified the 5, 23, 78, and 144 CpG marker sets used for prediction modelling by Liu et al*.* and therefore here as well. However, since these data were used in the initial marker discovery EWAS, we cannot exclude an overestimation effect in our prediction accuracy estimates obtained from these two cohorts (see below).

**Fig. 1:**
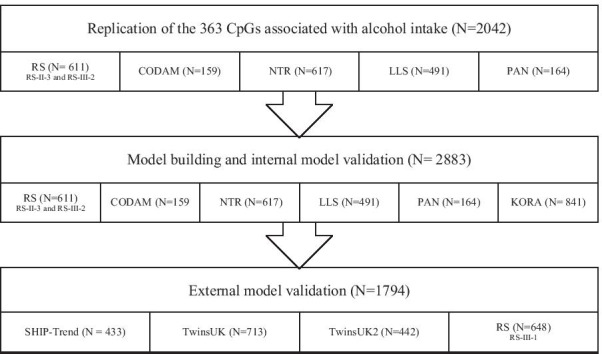
Use of study populations in each analysis. The 363 alcohol-associated CpGs previously identified by Liu et al*.* were replicated using data from 2042 participants of five cohorts studies embedded within the BIOS consortium. An additional 841 participants from the KORA F4 study were combined with these 2042 participants and together comprise our model building dataset. The model building dataset was used to train the prediction models and to test the reproducibility of the prediction models via internal cross-validation. The transportability of the models was tested in the external validation phase based on 1794 participants from three cohorts that were independent from the data used for model building and internal validation. Abbreviations: CODAM, Cohort on Diabetes and Atherosclerosis Maastricht; KORA, Cooperative Health Research in the Region of Augsburg study; LLS, Leiden Longevity Study; NTR, Netherlands Twin Register; PAN, Prospective ALS Study Netherlands; RS, Rotterdam Study; SHIP-Trend, Study of Health in Pomerania-Trend; TwinsUK- The TwinsUK Study; TwinsUK2- Subset of the TwinsUK Study

An overview of the datasets included in each analysis step of our study is provided in Fig. 1, their characteristics are summarized in Table 1 and described in detail in Additional file 1: Supplementary Methods.

**Table 1:** Dataset characteristics used in model building, internal and external validation

Study	N	Age (years), mean (SD)	Men (%)	BMI mean (SD)	Alcohol gr/day, Median (min, max)	Non-drinkers (%)	Light drinkers (%)	At-risk drinkers (%)	Heavy drinkers (%)
*Model building and internal validation dataset*									
RS-II-3/III-2	611	67 (6)	275 (45)	27.8 (4)	8.6 (1, 57)	0 (0)	545 (89)	52 (9)	14 (2)
CODAM	159	66 (7)	86 (54)	28.9 (4)	7.9 (0, 72)	12 (8)	117 (74)	23 (14)	7 (4)
NTR	617	39 (14)	188 (31)	24.6 (4)	5.1 (0, 69)	195 (32)	348 (56)	44 (7)	30 (5)
LLS	491	58 (6)	231 (47)	25.3 (3)	13.0 (0, 90)	36 (7)	309 (63)	98 (20)	49 (10)
PAN	164	62 (9)	100 (61)	26.0 (4)	11.0 (0, 77)	1 (1)	127 (77)	20 (12)	16 (10)
KORA F4	841	61 (9)	415 (49)	28.0 (5)	7.6 (0, 150)	251 (30)	354 (42)	133 (16)	103 (12)
Total dataset	2883	57 (14)	1295 (45)	26.7 (4)	8.0 (0, 150)	495 (17)	1800 (62)	370 (13)	218 (8)
*External validation datasets*									
SHIP-Trend	433	51 (14)	205 (47)	27.2 (4.1)	3.6 (0, 82)	47 (11)	346 (80)	28 (6)	12 (3)
TwinsUK	713	58 (10)	0 (0)	26.7 (5)	2.3 (0, 101)	187 (26)	423 (59)	67 (9)	36 (5)
TwinsUK2	442	59 (9)	0 (0)	26.6 (5)	5.3 (0, 94)	36 (8)	311 (70)	46 (10)	49 (11)
RS-III-1	648	59.6 (8)	298 (46)	27.7 (5)	6.4 (0, 57)	64 (10)	495 (76)	79 (12)	10 (2)

The total model building dataset was also used for internal ten-fold cross-validation. BMI, body mass index; CODAM, Cohort on Diabetes and Atherosclerosis Maastricht; KORA F4, The Cooperative Health Research in the Region of Augsburg study; LLS, Leiden Longevity Study; NTR, Netherlands Twin Register; PAN, Prospective ALS Study Netherlands; RS, Rotterdam Study; SD, standard deviation; SHIP, Study of Health in Pomerania-Trend cohort; TwinsUK, The TwinsUK Study; TwinsUK2, Subset of the TwinsUK Study. The alcohol categories were defined as; non-drinkers were defined as participants with no alcohol consumption; light drinkers with an alcohol consumption of 0 < g per day ⩽28 in men and 0 < g per day ⩽14 in women; and heavy drinkers with an alcohol consumption of ⩾42 g per day in men and ⩾28 g per day in women

### Replication of alcohol consumption associations

We aimed at replicating the association between alcohol consumption and the 363 CpGs previously identified by Liu et al. (*P* < 1 × 10^–7^) [12], using data from the BIOS consortium (*N* = 2042), which does not overlap with the Liu et al*.* data. This analysis revealed successful replication of 106 (29%) of these 363 CpGs after applying the Bonferroni-corrected significance threshold of *P* < 1.4 × 10^−4^ (0.05/363) and 283 (78%) CpGs based on the uncorrected nominal significance threshold of *P* < 0.05. All but one (cg06603309) of the 106 CpGs replicated after Bonferroni correction showed an inverse relationship with alcohol consumption, i.e., lower DNA methylation levels were associated with higher alcohol consumption, in line with the findings from the initial discovery EWAS by Liu et al. [12].

The top CpG in the Liu et al. discovery EWAS was cg02583484, annotated to the heterogeneous nuclear ribonucleoprotein A1 gene (*P* = 1.50 × 10^–19^, *β* = -0.0004), which was replicated in our independent dataset with a P-value of 1.16 × 10^–13^ and β = -0.0055. In our dataset, this CpG had a methylation range with a minimum DNA methylation beta-value of 0.1457 and a maximum of 0.4189. However, this marker was not among the 144 CpGs used by Liu et al*.* for inference and thus was not used by us for prediction validation (see below). Out of the 144 predictive CpGs, we replicated 29 CpGs (*P* < 1.4 × 10^−4^), which were all included in the 144-CpG model, 19 in the 78-CpG model, 6 in the 23-CpG model, and 3 in the 5-CpG model. A summary of the results is presented in Additional file 2: Table S1.

A total of 77 genes were annotated to the 106 replicated CpGs after Bonferroni correction. Gene ontology enrichment analysis via http://geneontology.org/page/go-enrichmentanalysis showed that these 77 genes were enriched in two biological processes. The ‘negative regulation of cellular macromolecule biosynthetic process’ included enrichment of 16 genes (3.49-fold, FDR = 4.91 × 10^–2^) and 25 genes were enriched in the ‘cellular response to chemical stimulus’ (2.63-fold, FDR = 3.80 × 10^–2^).

### Internal validation of alcohol consumption prediction models

**Fig. 2:**
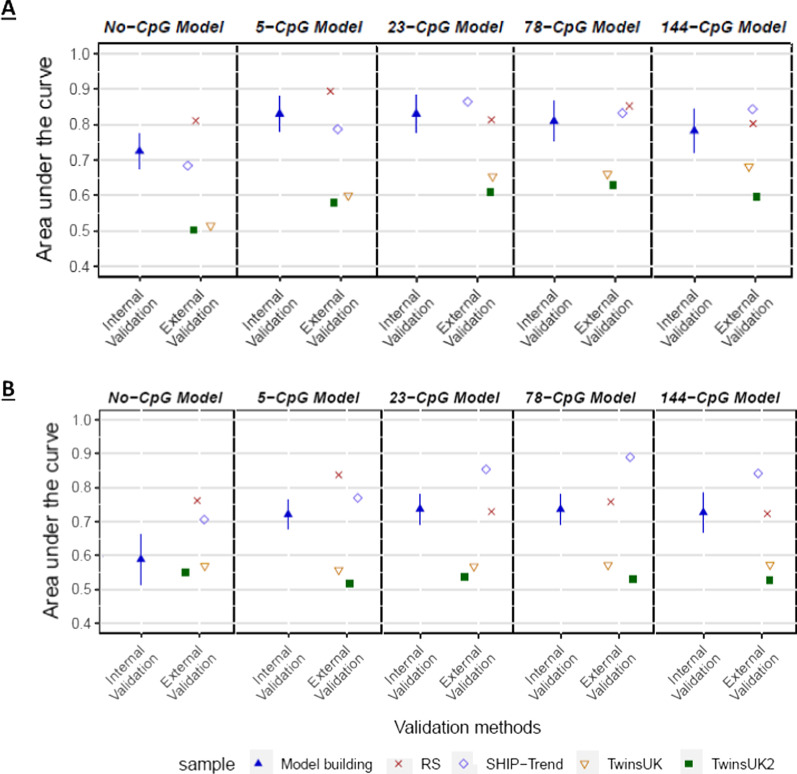
Epigenetic inference of alcohol consumption from blood based on Liu et al*.* biomarkers and models. Prediction accuracy for alcohol consumption expressed as Area Under the Curve (AUC) for **A** heavy drinkers vs. non-drinkers and **B** heavy drinkers vs. light drinkers using the CpG marker sets from Liu et al*.* [12]. Data from participants who do not fit the inferred categories were excluded from the respective prediction models following the approach used by Liu et al. ‘Internal Validation’: Mean AUC and SD from internal validation using ten-fold cross-validation in our model building dataset. ‘External Validation’: AUCs from external validation by applying our models trained in the model building dataset to independent data from three external validation cohorts (Rotterdam Study, *N* = 648; SHIP-Trend, *N* = 433; and TwinsUK, *N* = 713 and *N* = 442). Based on interview or self-reported information, non-drinkers were defined as participants with no alcohol consumption; light drinkers with an alcohol consumption of 0 < g per day ⩽28 in men and 0 < g per day ⩽14 in women; and heavy drinkers with an alcohol consumption of ⩾42 g per day in men and ⩾28 g per day in women. Abbreviations: RS- The Rotterdam Study; SHIP- Study of Health in Pomerania-Trend cohort; TwinsUK- The TwinsUK Study; TwinsUK2- Subset of the TwinsUK Study

To test the reproducibility of the seven prediction models reported by Liu et al*.* [12], we performed internal validation in our model building dataset via ten-fold cross-validation. The CpGs included per marker set and their average DNA methylation β-value per alcohol consumption category are presented in Additional file 3: Table S2. The mean AUC ± SD obtained by the ten logistic regression models are denoted as ‘Internal Validation’ in Fig. 2, Additional file 4: Figures S1-S5 and Additional file 5: Tables S3-S9. The highest mean AUC of 0.83 ± 0.05 was obtained for both the 5 and 23-CpG model for heavy drinkers vs. non-drinkers (Fig. 2A and Supplemental Table S3). For the other six models, we obtained for all marker sets an average AUC ≤ 0.75 in three models and ≤ 0.70 in the other three models (Fig. 2). Among all predictive marker sets, the lowest AUC of 0.61 ± 0.04 was obtained for the 144-CpG model for light drinkers vs. non-drinkers (Supplemental Table S9 and Supplemental Figure S5). In all seven prediction models, we obtained lower mean AUCs based on 144-CpGs compared to the models with lower numbers of CpG predictors. For example, the 144-CpG model for heavy drinkers vs. non-drinkers yielded an AUC of 0.78 ± 0.06 compared to 0.83 ± 0.05 obtained in the 5 and 23-CpG models (Fig. 2A and Supplemental Table S3). Similar results were obtained in the other models, and for some of the 78-CpG models, as shown in Additional file 4: Figures S1-S5 and Additional file 5: Tables S3-S9. Notably, these findings contrasts with that of Liu et al*.*, who reported increased prediction accuracies with increased numbers of CpG predictors [12].

### External validation of alcohol consumption prediction models

Aiming to test the transportability of the prediction models trained in our complete model building dataset (*N* = 2883), we performed external validation using data from three European cohorts (*N* = 1794) not considered for model building and internal validation: the Rotterdam study (RS-III-1), SHIP-Trend, and two datasets from the TwinsUK study. The obtained AUCs are denoted as ‘External Validation’ in Fig. 2, Additional file 4: Figures S1-S5 and Additional file 5: Tables S3-S9. The AUCs obtained from external validation varied strongly per model between the external validation datasets and differed with those obtained in the internal cross-validation. For example, the 144-CpG model for the heavy vs. non-drinkers yielded an AUC of 0.80 in RS and 0.84 in SHIP-Trend, while in TwinsUK and TwinsUK2 they were considerably lower with 0.68 and 0.60, respectively, and the mean AUC in the internal cross-validation was 0.78 ± 0.06 (Fig. 2A and Additional file 5: Tables S3). Similarly, the 23-CpG heavy vs. non-drinker model yielded AUCs of 0.81, 0.87, 0.65, 0.61, and 0.83 ± 0.05, respectively. The high variance between obtained AUCs in the different external validation datasets was also observed in several other models as shown in Additional file 4: Figures S1-S5 and Additional file 5: Tables S3-S9. The high AUC variance we observed in the external validation between datasets indicate non-robust performance of these prediction models, when applied to independent datasets.

### New models for epigenetic inference of alcohol consumption using all categories

**Fig. 3:**
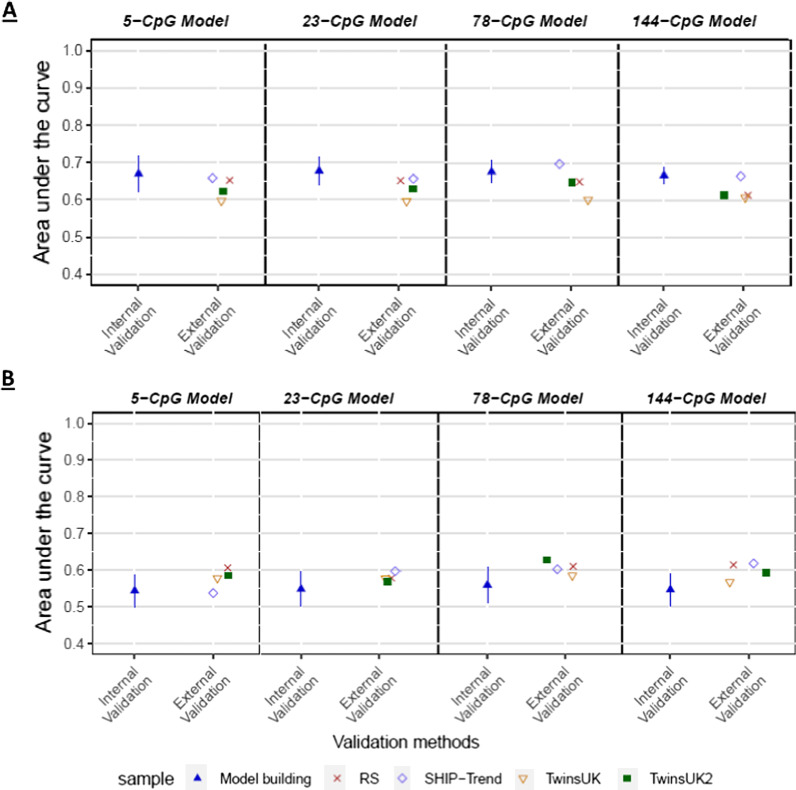
Epigenetic inference of alcohol consumption from blood based on newly developed models including all categories. Prediction accuracy for alcohol consumption expressed as Area Under the Curve (AUC) for **A** heavy and at-risk drinkers vs. light and non-drinkers and **B** heavy, at-risk and light drinkers vs. non-drinkers. In these models, all available participants from all categories were included, in contrast to Fig. 2. ‘Internal Validation’: Mean AUC and SD from internal validation using ten-fold cross-validation in our model building data set. ‘External Validation’: AUCs from external validation by applying our model trained in the model building dataset to independent data from three external validation cohorts (Rotterdam Study, *N* = 648; SHIP-Trend, *N* = 433; and TwinsUK, *N* = 713 and *N* = 442). For phenotype definition, see legend of Fig. 2. Abbreviations: RS- The Rotterdam Study; SHIP- Study of Health in Pomerania-Trend cohort; TwinsUK- The TwinsUK Study; TwinsUK2- Subset of the TwinsUK Study

Finally, we developed two new models for epigenetic inference of alcohol consumption from blood by considering all data from all individuals of all four alcohol consumption categories in our prediction models, thereby refraining from excluding categories from prediction modeling as was done by Liu et al. [12]. To this end, we used all individuals from the model building dataset to build and internally validate via ten-fold cross-validation the models, as well as all individuals from our external validation datasets to externally validate the models. This was done for two different models. Model 1 comprised all heavy and at-risk drinkers combined vs. all light and non-drinkers combined. Model 2 included all heavy, at-risk, and light drinkers combined (i.e., all drinkers no matter the level of alcohol consumption) vs. all non-drinkers. The average AUCs ± SDs from internal cross-validation in the model building dataset were denoted as ‘Internal Validation’ and the four AUCs from the four external validation datasets as ‘External Validation’ (Fig. 3 and Additional file 5: Tables S10 and S11).

Regarding model 1 for inferring heavy and at-risk vs. light and non-drinkers, the (mean) AUCs from internal cross-validation and from external validations ranged between 0.67–0.68 and 0.60–0.70, respectively across all marker sets (Fig. 3A and Additional file 5: Table S10). Regarding model 2 for inferring all drinkers (heavy plus risk plus light) vs. non-drinkers, the AUCs from the two validation approaches based on the 5-CpG and the 23-CpG models were between 0.54–0.55 and 0.54–0.61, respectively. For the 78-CpG and the 144-CpG models, similarly low AUCs were seen in the internal validation, between 0.55–0.56, with slightly higher AUCs in the external validation datasets, between 0.57–0.63 (Fig. 3B and Additional file 5: Table S11). Thus, compared to the Liu et al*.* models based on an approach that leaves out data, the new models based on all data achieved generally lower AUCs, while the AUC variance in the external validation was much less pronounced between the datasets than observed for the Liu et al. models (Fig. 2, Additional file 4: Figures S1-S5, and Additional file 5: Table S3-S9).

## Discussion

In this study, we firstly performed replication analysis in an independent dataset of the EWAS results on alcohol consumption previously reported by Liu et al*.* [12], which delivered Bonferroni-corrected significant replication of close to one-third of the previously identified CpGs. Our smaller sample size of 2042 compared to 9642 in the Liu et al*.* study might be the reason why we only replicated one-third of the previously identified CpGs. However, using the nominal significance threshold (*P* < 0.05), we replicated the association of close to 80% of these CpGs.

Secondly, by using data from eight population-based cohorts, we performed in-depth validation of the biomarkers and models reported by Liu et al. [12] to infer alcohol consumption from blood. Reproducibility assesses the degree to which the model fits the real patterns rather than random noise in the data [28]. To test for reproducibility of the models, we performed internal model validation by implementing a ten-fold cross-validation scheme. The heavy vs. non-drinkers model obtained the highest average AUCs of the seven models in the cross-validation. Interestingly, the 144 and 78-CpG models obtained a lower average AUC than the 5 and 23-CpG models. In addition, in all models, we observed a higher AUC for the models including less CpGs compared to the 144-CpG model and to some extent also for the 78-CpG models. In contrast, Liu et al*.* reported increased prediction accuracies for models with increased number of CpG predictors [12]. Our findings provide evidence that these 144-CpG models are over-fitted and thus, likely not reproducible. This increased risk for overfitting by including an increasing number of CpGs was also suggested by Hattab et al*.* [16]. Overfitting of a model is more likely to be observed when the ratio of the number of variables to the number of samples is small [29]. In this context, Harrell et al. [30] suggested that for generalizable binary models, no more than one predictor per ten participants in the smallest outcome category should be examined when fitting a regression model. As some of the findings of the analysis come from partially fitting to the noise on top of the true signal, noise features may be assigned nonzero coefficients due to chance associations with response to the training set [31]. Overall, the AUCs we achieved via internal cross-validation for the different models and marker sets were considerably lower than those reported by Liu et al*.* [12]*.* Also, the results obtained in our internal validation were much lower compared to the results we obtained when applying the same methods as Liu et al*.*, e.g. training and testing the model in the same dataset, in our model building dataset (see Additional file 6 for results). This confirms previous conclusions [16] that the prediction accuracies reported by Liu et al*.* represent overestimates.

The transportability of the prediction models was tested by applying the models (trained in the model building dataset) to four validation datasets from three cohorts. Three models yielded an AUC ≤ 0.75 in the internal validation and in all four external validation datasets across all marker sets. For the other four models, a large variability in AUCs was obtained between the different datasets. Overall, in these four models, we obtained similar to higher AUCs for the Rotterdam Study and SHIP-Trend compared to the internal validation, while both TwinsUK datasets provided lower AUCs than the internal validation. It is important to note that the datasets from the Rotterdam Study and the TwinsUK (*N* = 713) were both included in the EWAS for predictive marker discovery by Liu et al*.* [12]. The use of the same participants here and by Liu et al*.* could have led to an overestimation of the prediction accuracies. Surprisingly, the AUCs we obtained in the TwinsUK (*N* = 713) in the current study are in most models much lower than the AUCs we obtain in SHIP-Trend and the Rotterdam study. The results obtained in the Rotterdam Study were overall more similar to those obtained by SHIP-Trend. These results suggest that the use of the same participant, here and by Liu et al*.,* did not positively impact the prediction accuracies obtained in our study. The subset of the TwinsUK (*N* = 442) includes re-processed DNA methylation data and a different FFQ-based approach for alcohol consumption information. Nevertheless, also in this dataset we obtain lower AUCs compared to the Rotterdam Study and SHIP-Trend, with very similar result as for the total TwinsUK (*N* = 713) dataset. Notably, the AUCs from external validation were generally lower than the AUCs reported by Liu et al*.* [12] and as the similarly high AUCs we obtained from our model building dataset, when applying the same methods as Liu et al. (see Additional file 6 for results), providing further evidence that the prediction accuracies reported by Liu et al*.* represent overestimates. This is in line with our conclusion from internal validation and as suggested by Hattab et al*.* [16].

Yousefi et al*.* [17] estimated DNA methylation-derived scores using the coefficients made available by Liu et al*.* [12] in participants of the Accessible Resource for Integrated Epigenomic Studies (ARIES) parental generation at midlife cohort (*N* = 1049, mean age = 50.2 ± 5.4 SD) as discovery dataset. A limitation of the study by Yousefi et al*.* [17] was the relatively small sample size in the higher alcohol consumption categories, with only 14 heavy drinkers and 67 at-risk drinkers. As a result, the lower AUCs obtained by Yousefi et al*.* [17] compared to Liu et al*.* [12] could possibly be due to the small sample size rather than an accurate representation of the true model prediction accuracies. In the current study, however, we have implemented 2883 participants, including 495 non-drinkers, 1800 light drinkers, 370 at-risk drinkers, and 218 heavy drinkers, with an age range of 19–87 years (mean age 57.4 ± 13.8 SD). By including more participants, especially in the categories with higher alcohol consumption, we overcome this possible sample size limitation and thus provide a more reliable representation of the models’ prediction accuracies. Yousefi et al*.* [17] obtained low AUCs from 0.48 to 0.57 to distinguish heavy drinkers vs. non-drinkers and 0.55 to 0.57 for heavy drinkers vs. light drinkers in adults at midlife. In our external validation, we obtained AUCs from 0.80 to 0.89 in the Rotterdam Study, 0.68 to 0.87 in SHIP-Trend, 0.52 to 0.68 in TwinsUK (*N* = 713), and 0.50–0.63 in TwinsUK2 (*N* = 442) for distinguishing heavy vs. non-drinkers and 0.72 to 0.84 in the Rotterdam Study, 0.71 to 0.89 in SHIP-trend, 0.56 to 0.57 in TwinsUK (*N* = 713), and 0.52–0.55 in TwinsUK2 (*N* = 442) for heavy drinkers vs. light drinkers. The results in the Rotterdam Study and SHIP-Trend are overall higher than those obtained by Yousefi et al*.*, while the results obtained in the TwinsUK are very similar to those obtained by Yousefi et al*.* In addition, the high variability in the obtained AUCs in our study and the close to random inference obtained by Yousefi et al*.* [17] study suggest that the tested CpGs are not as suitable as previously suggested for achieving transportable and accurate alcohol consumption prediction models.

The exclusion from prediction modelling of data from participants who did not fit the inferred categories, as done by Liu et al*.* [12], means that such models cannot be applied to the general population, where individuals with the excluded categories exist and can never be inferred correctly because their category was not considered in the prediction model. Therefore, for a prediction models to be applicable in cohort studies used in epidemiology research, or any practical applications in the clinic and beyond, should be designed in data that realistically reflects the general population. For that reason, we have developed two additional models in which data from all individuals of all alcohol categories were included and validated them internally via cross-validation as well as externally in independent datasets. The first model for heavy and at-risk drinkers vs. light and non-drinkers provided cross-validated AUCs between 0.67 and 0.68 across all four CpG marker sets. These results are close to the lower 95% CI of the 450-CpG based model previously developed by McCartney et al. [15], which had an AUC of 0.73 (95% CI = 0.69–0.78) to distinguish light-to-moderate drinkers from heavy drinkers. Four CpGs overlap between this 450-CpG model and the 23-CpG model: cg00252472, cg06690548, cg11613559, and cg12825509. In addition, two more CpGs overlap with the 144-CpG model; cg11376147 and cg18032812. In the external validation, we obtained AUCs in the range of 0.60–0.70 across all marker sets and all external validation cohorts. In the second model, which distinguishes heavy, at-risk, and light drinkers vs. non-drinkers, we obtained AUCs at 0.54–0.63 in both internal and external validation. Thus, when applying appropriate prediction methodology by not excluding participant data and performing external validation, the CpG marker sets reported by Liu et al*.* [12] yield much lower prediction accuracies as compared to the AUCs previously published and obtained here based on the previous approach.

Our study has strengths and limitations that should be considered when interpreting the results. The main strengths of our study are the use of a large dataset from several cohorts with similar numbers for the different categories as Liu et al*.* [12], and the use of four datasets for external model validation. Moreover, our findings agree with a previous validation study based on a different methodology [17], while our larger dataset improved the limitations of the limited data used in the previous validation study. The main limitation of our study, as well as in the previous studies, is that the alcohol consumption information is based on interviews or self-reported questionnaires, which are generally considered unreliable in terms of underestimating actual alcohol consumption. Regarding the putative inaccuracy of interviews and self-reported alcohol consumption used here as phenotypes, we cannot know how error-prone these reports are. In particular, it is possible that heavy drinkers might not be able to or might be hesitant or unwilling to accurately recall or report their high alcohol consumption. Also, there is variability in the questionnaires regarding the reference time window. For example, KORA participants were asked about alcohol consumption in the past few days, which may or may not be representative of the participants’ long-term alcohol consumption habits. Also, non-drinkers may include lifetime non-drinkers but also sober alcoholics; however, it is not yet clear how this could affect the obtained DNA methylation patterns. Because all available studies, including the EWAS that identified CpGs associated with alcohol consumption, used interviews or self-reported alcohol consumption information, this is a general limitation that cannot be easily solved, as methods to empirically measure alcohol concentrations are not suitable for estimating long-term alcohol consumption. Another source of uncertainty may lie in the calculation for alcohol consumption in grams/day, which presents a slight variation in the formula used between the different cohorts. The variation in alcohol consumption data collection between the cohorts might also play a role in the variance we obtain in the prediction AUCs.

Another shortcoming of our study was the inclusion of only participants from European ancestry. As DNA methylation patterns might differ between populations [32], the absence of non-European participants during marker discovery and model building might prohibit accurate model transportability to non-European populations. Hence, future studies would benefit from a trans-ethnic prediction marker discovery, model building, and validation.

Overall, our extensive validation testing of the different CpG sets reported by Liu et al. [12] for inferring alcohol consumption from blood demonstrates that using appropriate prediction methodology regarding both separating datasets for model building and model testing by performing internal cross-validation and external validation, and including all alcohol consumption categories and individuals in the prediction modelling, yields much lower prediction accuracies and with a high variance between validation cohorts for the Liu et al. models as were previously published. This allows us to conclude that the currently available DNA methylation predictors for alcohol consumption need to be improved considerably before epigenetic inference of alcohol consumption from blood can be considered for practical applications in the clinic and beyond. Our study implies that, currently, we are far away from epigenetic inference of alcohol consumption from blood in research and practical applications, despite EWASs having already delivered hundreds of associated CpGs. Thus, further EWASs on alcohol consumption are necessary to increase the number of associated CpGs, including replication studies for the identified CpGs. Established CpGs replicated in several independent studies could provide better predictive markers than CpGs identified in one large meta-analysis. These CpGs will need to be carefully tested for their value to improve the low accuracy in inferring alcohol consumption from blood achieved with the currently available marker sets.

## Methods

### Study populations

This study was embedded within the Biobank-based Integrative Omics Study (BIOS) consortium [26], by including participants from the Rotterdam Study (sub-cohorts RS-II-3 and RS-III-2) (*N* = 611) [18], Cohort on Diabetes and Atherosclerosis Maastricht (CODAM) (*N* = 159) [19], the Netherlands Twin Register (NTR) (*N* = 617) [20], the Leiden Longevity Study (LLS) (*N* = 491) [21], and the Prospective ALS Study Netherlands (PAN) (*N* = 164) [22]. Additionally, we included 841 participants from The Cooperative Health Research in the Region of Augsburg (KORA) study (F4) [23]. External validation was conducted in independent samples (i.e., not used for model building and internal validation) from the Study of Health in Pomerania (SHIP)-Trend cohort (*N* = 433) [24], two datasets from the TwinsUK Study with overlapping participants; TwinsUK (*N* = 713) and TwinsUK2 (*N* = 442) [25], and participants from the Rotterdam Study sub-cohort RS-III-1 (*N* = 648) that are not included in the BIOS consortium. Alcohol consumption information was obtained via interviews or self-reported questionnaires. Cohort-specific data collection and dataset characteristics are summarized in Table **1** and described in detail in the Supplementary Methods (Additional file 1).

### Microarray-based DNA methylation quantification

DNA was extracted from whole peripheral blood and analyzed with the Illumina Infinium Human Methylation 450 K BeadChip (Illumina Inc, San Diego, CA, USA) or the Infinium MethylationEPIC BeadChip (Illumina Inc, San Diego, CA, USA) to obtain the DNA methylation measurements. Details on cohort-specific methods are provided in Supplementary Methods (Additional file 1). The methylation proportion of a CpG site was reported as the methylation β-value in the range of 0 to 1.

### Candidate-CpG association study for alcohol consumption and gene annotation

Using the data from the BIOS Consortium (*N* = 2042), we tested the association of the 363 CpGs previously found to be significantly associated (*P* < 1 × 10^–7^) with alcohol consumption [12]. Alcohol consumption levels (grams/day) were right-skewed and contained non-drinkers; therefore, the log-transformed alcohol consumption (log (g per day + 1)) was used as the independent variable. The β-values of the 363 CpGs were included as the dependent variable and the analysis was adjusted for age, sex, BMI, batch effects (plate, plate location, and cohort ID), and Houseman-imputed white blood cell counts (WBC) for CD4T cells, CD8T cells, natural killer cells, B-cells, granulocytes, and monocytes [33]. The Bonferroni multiple-test corrected 5% significance level of *P* < 1.4 × 10^−4^ (0.05/363) was applied. All analyses were performed using the statistical package R, version 3.4.3.

We obtained the genes annotated to the replicated CpGs using the annotation file provided by Illumina and performed Gene ontology (http://geneontology.org/page/go-enrichmentanalysis) enrichment analysis for these genes.

### Validation of the previously published prediction models

The BIOS and KORA DNA methylation data were combined as the model building dataset (*N* = 2883) using the “ComBat” function [34] (R-package “sva” [35]) to adjust for the known batches via an empirical Bayesian framework adjusting for age and sex. Then, possible confounders were regressed out using linear regression models, obtaining the residuals for each CpG adjusted for age, sex, BMI, batch effects (plate, plate location, and cohort ID), and Houseman-imputed WBC (CpG = age + sex + BMI + batch effects + WBC).

The self-reported phenotypic data on alcohol consumption were categorized according to their alcohol consumption levels for which we used the same cut-off categories as described by Liu et al. [12], to allow for direct comparison of the models’ performance; non-drinkers: participants with no alcohol consumption; light drinkers: participants with alcohol consumption of 0 < g per day ≤ 28 in men and 0 < g per day ≤ 14 in women; at-risk drinkers: participants with alcohol consumption of 28 < g per day < 42 in men and 14 < g per day < 28 in women; heavy drinkers: participants with alcohol consumption of ≥ 42 g per day in men and ≥ 28 g per day in women.

The alcohol categories used in each model were inferred using the same seven prediction models as previously applied by Liu et al*.* with heavy drinkers vs. all other categories separately, i.e., heavy drinkers vs. (1) non-drinkers, (2) light drinkers, (3) pooled individuals of light or non-drinkers, (4) at-risk drinkers, as well as two-category combinations between the other categories including (5) at-risk drinkers vs. non-drinkers, (6) at-risk drinkers vs. light drinkers, and (7) light drinkers vs. non-drinkers. In all models, the former category was the ‘cases’ (coded as “1”) and the latter was the ‘control’ group (coded as “0”). Selecting a subset of categories in prediction modeling and AUC estimation, as was done by Liu et al*.,* may limit the possibility to extrapolate the result to the general population. Hence, we replicated this approach solely for outcome compatibility reasons. All seven models were trained for the null model, which only includes age, sex, and BMI, and subsequently the null model combined with the residuals of the four CpG sets (5, 23, 78, or 144 CpGs). The CpGs included per model and the average DNA methylation β-values per category are presented in Additional file 3: Table S2.

### Internal and external validation of the previously published prediction models

We tested the reproducibility (internal validation) and transportability (external validation) of the prediction models conducted by Liu et al*.* [12, 28]. First, we adopted a tenfold cross-validation scheme [36] in which the whole model building dataset (*N* = 2883) was randomly distributed into ten non-overlapping subsets. The logistic regression model was trained in a combination of nine subsets (90% of the data), which was then applied to the remaining subset (10% of the data) to infer the participants’ alcohol status. This method results in ten different training (90%) and testing (10%) sets. We trained the seven models in the training sets (90%) using binomial regression analysis with the alcohol categories (coded as 1/0) as the dependent variable and age, sex, and BMI without (the null model) or with a set of (the residuals of the) CpGs as the independent variables (Alcohol category = age + sex + BMI (+ ResCpGs_5, 23, 78, 144_)). For this purpose, the “glm” function with “binomial” as family and “logit” as link were used. The models were then applied to the test set (10%) using the “predict” function. The prediction performance of the models was assessed using “roc” (R-package “pROC”) that calculates the AUC per model. This method resulted in ten logistic regression models and consequently, ten AUCs from which average values were estimated and standard deviation were obtained.

Secondly, we externally validated the models that were trained in the complete model building dataset (*N* = 2883) by testing them in four external validation datasets, using the “predict” function. The “roc” function (R-package “pROC”) was again used to calculate the AUC per model. The independent cohorts used our previously described pre-processing procedure by regressing out the potential covariates. The TwinsUK study used a linear mixed model to additionally adjust for twin family structure and zygosity using random effects. Also, sex was not included in the pre-processing steps because solely women were included in the TwinsUK analysis. Notably, according to the above-described scenarios, both internal and external validations followed the same approach previously applied by Liu et al*.* [12] in that individuals not fitting the inferred categories were excluded from the prediction analysis.

### Prediction modeling without excluding categories and data

Finally, we trained as well as internally and externally validated two new prediction models comprising all individuals in the prediction modeling, i.e., (1) heavy and at-risk drinkers vs. light and non-drinkers and (2) heavy, at-risk and light drinkers (i.e. all drinkers no matter how much) vs. non-drinkers. These two models were internally validated via tenfold cross-validation and externally validated in four datasets. As age, sex, and BMI are already accounted for in the residuals we solely included the four CpG marker sets in these models. The coefficients for these models are presented in Additional file 7: Table S21.

## Supplementary Information

**Additional file 1: Supplementary methods**. Comprising the study cohort characteristics.

**Additional file 2: Table S1**. Which shows the replication results for the candidate-CpG association study on alcohol consumption.

**Additional file 3: Table S2**. Presenting the CpGs incorporated in the four prediction marker sets, together with the mean DNA methylation residual β-value per alcohol consumption category per CpG in the model building dataset, and in the four external validation datasets.

**Additional file 4: Figures S1–S5**. **Figure S1** shows the results for heavy drinkers vs. light and non-drinkers; **Figure S2** shows the results for heavy drinkers vs. at-risk drinkers; **Figure S3** shows the results for at-risk drinkers vs. non-drinkers; **Figure S4** shows the results for at-risk drinkers vs. light drinkers; and **Figure S5** shows the results for light drinkers vs. non-drinkers.

**Additional file 5: Tables S3–S11**. **Table S3** shows the results for heavy drinkers vs. non-drinkers; **Table S4** shows the results for heavy drinkers vs. light drinkers; **Table S5** shows the results for heavy drinkers vs. light and non-drinkers; **Table S6** shows the results for heavy drinkers vs. at-risk drinkers; **Table S7** shows the results for at-risk drinkers vs. non-drinkers; **Table S8** shows the results for at-risk drinkers vs. light drinkers; **Table S9** shows the results for light drinkers vs. non-drinkers; **Table S10** shows the results for heavy and at-risk drinkers vs. light and non-drinkers; and **Table S11** shows the results for heavy, at-risk and light drinkers vs. non-drinker.

**Additional file 6**. We provide the data, methods, and results regarding the replication of the Liu et al. approach; including accompanying supplementary Tables S12–S20, and supplementary Figures S6–S14. **Table S12** shows the results for heavy drinkers vs. non-drinkers; **Table S13** shows the results for heavy drinkers vs. light drinkers; **Table S14** shows the results for heavy drinkers vs. light and non-drinkers; **Table S15** shows the results for heavy drinkers vs. at-risk drinkers; **Table S16** shows the results for at-risk drinkers vs. non-drinkers; **Table S17** shows the results for at-risk drinkers vs. light drinkers; **Table S18** shows the results for light drinkers vs. non-drinkers; **Table S19** shows the results for heavy and at-risk drinkers vs. light and non-drinkers; and **Table S20** shows the results for heavy, at-risk and light drinkers vs. non-drinker. **Figure S6** shows the results for heavy drinkers vs. non-drinkers; **Figure S7** shows the results for heavy drinkers vs. light drinkers; **Figure S8** shows the results for heavy drinkers vs. light and non-drinkers; **Figure S9** shows the results for heavy drinkers vs. at-risk drinkers; **Figure S10** shows the results for at-risk drinkers vs. non-drinkers; **Figure S11** shows the results for at-risk drinkers vs. light drinkers; **Figure S12** shows the results for light drinkers vs. non-drinkers; **Figure S13** shows the results for heavy and at-risk drinkers vs. light and non-drinkers; and **Figure S14** shows the results for heavy, at-risk and light drinkers vs. non-drinker.

**Additional file 7: Tables S21**. Providing the coefficients for our two new models including four-categories for each marker set.

**Additional file 8.** List of all authors included in the BIOS consortium.

## Data Availability

The data from the BIOS Consortium supporting the conclusions of this article are available from the European Genome-phenome Archive (EGA) under accession number EGAC00001000277. Data from the Rotterdam Study can be requested at http://www.epib.nl/research/ergo.htm or contact M. Arfan Ikram (m.a.ikram@erasmusmc.nl). The informed consents given by KORA study participants do not cover data posting in public databases. However, data are available upon request from KORA Project Application Self-Service Tool (https://epi.helmholtz-muenchen.de/). Data requests can be submitted online and are subject to approval by the KORA Board. The data of the SHIP study cannot be made publically available due to the informed consent of the study participants, but it can be accessed through a data application form available at https://fvcm.med.uni-greifswald.de/ for researchers who meet the criteria for access to confidential data. The TwinsUK methylation dataset can be applied for through the TwinsUK data access procedures (described in detail at https://twinsuk.ac.uk/resources-for-researchers/access-our-data/).

## Abbreviations

ARIC: The Atherosclerosis Risk in Communities study
ARIES: Accessible Resource for Integrated Epigenomic Studies
AUC: Area under the curve
BIOS Consortium: Biobank-based Integrative Omics Study Consortium
BMI: Body mass index
CODAM: Cohort on Diabetes and Atherosclerosis Maastricht
DALYs: Disability-adjusted life-years
EWAS: Epigenome-wide association study
FHS: The Framingham Heart Study
KORA study: The Cooperative Health Research in the Region of Augsburg
LBC1936: The Lothian Birth Cohort 1936
LLS: Leiden Longevity Study
MESA: The Multi-Ethnic Study of Atherosclerosis
NTR: The Netherlands Twin Register
PAN: Prospective ALS Study Netherlands
SD: Standard deviation
SHIP-Trend: Study of Health in Pomerania-Trend
vs.: Versus
WBC: White blood cell counts
