# Supplementary material for: Validating biomarkers and models for epigenetic inference of alcohol consumption from blood
Source: Clin Epigenetics. 2021 Oct 26;13:198. doi: 10.1186/s13148-021-01186-3 (PMC8549335; doi:10.1186/s13148-021-01186-3)
Supplement: Supplementary file 1 — Additional file 1: Supplementary methods. Comprising the study cohort characteristics. [file 13148_2021_1186_MOESM1_ESM.pdf]

Additional file 1: Supplementary Methods

This study was embedded within the Biobank-based Integrative Omics Study (BIOS) Consortium [1], using data from five Dutch cohorts namely: the Rotterdam Study, the Cohort on Diabetes and Atherosclerosis Maastricht, The Netherlands Twin Register, the Leiden Longevity Study, and the Prospective ALS Study Netherlands. Additionally, we included participants from The Cooperative Health Research in the Region of Augsburg study (F4), the Study of Health in Pomerania-Trend cohort, and the TwinsUK study.

The Rotterdam Study (RS) [2] is a population-based cohort study that aims to unravel etiology, preclinical course, natural history and potential targets for intervention for chronic diseases in mid-life and late-life. All residents of Ommoord, a district of Rotterdam in the Netherlands, aged 55 years and older were invited in 1990 to participate (RS-I). An additional 3011 participants, who had reached the age 55 years or who were 55 years and over and had moved into the research area, were included in 2000 (RS-II). In 2006, a third cohort of 3934 participants aged 45 years and older was initiated (RS-III). As of 2008, the Rotterdam Study cohort includes a total of 14926 participants. In our analysis, only participants with complete information on alcohol intake and DNA methylation data on the 144 predictive CpGs were included. This resulted in the inclusion of 611 participants from visits RS-II-3 and RS-III-2 as part of the BIOS Consortium and 648 participants from RS-III-1. Information on habitual alcohol consumption in the past year was obtained during home interviews and the continuous alcohol phenotype “grams per day” was calculated by multiplying the average number of glasses consumed per day by 10 according to Dutch standard glasses.

Acknowledgment

The Rotterdam Study is funded by Erasmus Medical Center and Erasmus University, Rotterdam, Netherlands Organization for the Health Research and Development (ZonMw), the Research Institute for Diseases in the Elderly (RIDE), the Ministry of Education, Culture and Science, the Ministry for Health, Welfare and Sports, the European Commission (DG XII), and the Municipality of Rotterdam. The authors are grateful to the Rotterdam Study participants, the staff involved with the Rotterdam

Study and the participating general practitioners and pharmacists.

The generation and management of the Illumina 450K methylation array data (EWAS data) for the Rotterdam Study was executed by the Human Genotyping Facility of the Genetic Laboratory of the Department of Internal Medicine, Erasmus MC, the Netherlands. The EWAS data was funded by the Genetic Laboratory of the Department of Internal Medicine, Erasmus MC, and by the Netherlands Organization for Scientific Research (NWO; project number 184021007) and made available as a Rainbow Project (RP3; BIOS) of the Biobanking and Biomolecular Research Infrastructure Netherlands (BBMRI-NL). We thank Mr. Michael Verbiest, Ms. Mila Jhamai, Ms. Sarah Higgins, Mr. Marijn Verkerk, and Lisette Stolk for their help in creating the methylation database. We thank Pascal Arp, Mila Jhamai, MarijnVerkerk, Lizbeth Herrera and Marjolein Peters for their help in creating the GWAS database.

The Cohort on Diabetes and Atherosclerosis Maastricht (CODAM) [3] consists of a selection of 547 subjects from a larger population-based cohort [4]. Participants were included into CODAM by a moderately increased risk of developing cardiometabolic diseases, of European ancestry and over 40 years of age and additionally met at least one of the following criteria: increased body mass index (BMI; >25), a positive family history for type 2 diabetes, a history of gestational diabetes and/or glycosuria, or use of antihypertensive medication. In the current study, 159 CODAM participants were available with data on alcohol intake and DNA methylation levels on the 144 predictive CpGs. Dietary intake was assessed using a self-administered 79-item semi-quantitative food frequency questionnaire. The alcohol consumption frequencies were converted to the continuous alcohol phenotype “grams per day” using an extended version of the Dutch Food Composition table from 2001 (NEVO).

Acknowledgment

CODAM was supported by grants of the Netherlands Organization for Scientific Research (940–35–034) and the Dutch Diabetes Research Foundation (98.901).

The Netherlands Twin Register (NTR) [5, 6] was established in 1987 to study the extent to which genetic and environmental influences cause phenotypic differences between individuals. The NTR has 120,000 twins and their relatives enrolled with a total of 255,729 registered participants as of 2019. To this end, data was collected with a focus on health, lifestyle, personality, brain development, cognition, mental health and aging. In the current study, 617 unrelated NTR participants were available with data on alcohol intake and DNA methylation levels on the 144 predictive CpGs. Food frequency questionnaires were used to obtain the number of glasses alcohol consumed per week. This value was divided by seven to obtain the number of glasses of alcohol per day, which was subsequently multiplied by 12 to come to the continuous alcohol phenotype “grams per day”.

Acknowledgment

The authors warmly thank all participants. Phenotype information was obtained from multiple NTR surveys (survey 2 through survey 10), which were funded by BBRMI-RP12; KNAW PAH/6635; BBRMI-CP2011-38; ERC Starting grant 284167; Efic Grunenthal Grant; ZonMW (Addiction) 31160008; Borderline Foundation; NWO-MW 904-61-193; METC 2001/069; NWO 985-10-002; NWO 904-61-090, METC 96/164; VU-USF 96/22).

This work was further supported by the European Union Seventh Framework Program (FP7/2007-2013) under grant agreement no 602768; the BBRMI-NL -financed BIOS Consortium (NWO 184.021.007) and the European Research Council (ERC-230374), the Royal Netherlands Academy of Science Professor Award (PAH/6635) and the NWO-funded X-omics project (184.034.019).

Leiden Longevity Study (LLS) [7, 8] was established to identify genetic factors influencing longevity and examine their interaction with the environment as a means to develop interventions to increase health at older ages. Long-lived siblings of European descent were recruited together with their offspring and their offspring's partners. There are four inclusion criteria for the long-living subjects: (1) men must be aged ≥89 years and women ≥ 91 years; (2) subjects must have at least one living sibling; (3) the sib pairs have an identical mother and father; (4) the parents of the sib-ship are

Dutch and Caucasian. In total, this led to the ascertainment of 944 long-lived siblings from 421 families, together with 1671 of their offspring and 744 partners. In the current study, 491 participants (unrelated offspring and their partners) were available with data on DNA methylation data and alcohol intake. Using a food frequency questionnaire, participants of the Leiden Longevity Study reported in 2006 the intake of foods consumed during the previous month [9]. The obtained data were converted into energy and nutrient intake by using the NEVO food composition database of 2006.

Acknowledgment

The LLS was supported by a grant from the Innovation-Oriented Research Program on Genomics (SenterNovem IGE01014 and IGE05007), the Centre for Medical Systems Biology and the National Institute for Healthy Ageing (Grant 05040202 and 05060810), all in the framework of the Netherlands Genomics Initiative/Netherlands Organization for Scientific Research.

Prospective ALS Study Netherlands (PAN) [10] is a population-based study performed in the Netherlands including patients above 15 years of age and diagnosed with suspected, possible, probable or definite ALS according to the El Escorial criteria and control samples. Prevalent cases were all cases diagnosed before 31 December 2008 and still alive at that date. Incident cases were identified from 1 January 2006 to 31 December 2009. In 2016, more than 3200 participants were included in this study. In the current study, 164 PAN participants were available with data on alcohol intake and DNA methylation levels on the 144 predictive CpGs. The food frequency questionnaire was used to obtain alcohol consumption and the continuous alcohol phenotype “grams per day” was obtained using the NEVO food composition database of 2006.

Acknowledgment

The PAN study is funded by the Stichting LS (www.als-stichting.nl).

The Cooperative Health Research in the Region of Augsburg (KORA) study is a series of independent population-based epidemiological surveys and follow-up studies of participants living in the region of Augsburg, Southern Germany. The KORA F4 study, a seven-year follow-up study of the

KORA S4 survey (examined 1999-2001), was conducted between 2006 and 2008. The standardized examinations applied in the survey have been described in detail elsewhere [11]. A total of 3080 subjects with ages ranging from 32 to 81 years participated in the examination. In a random subgroup of 1802 KORA F4 subjects the genome-wide DNA methylation patterns were analysed. In the current study, 841 KORA participants were available with data on alcohol intake and DNA methylation levels on the 144 predictive CpGs. Alcohol consumption was measured by questionnaires on drinking of beer, light beer, wine, spirits, and mixed drinks during the previous weekend (Saturday and Sunday) and the day before the last working day. The continuous phenotype, “grams per day”, is calculated using the following converters between drinks and grams: one liter beer was equivalent to 40.0 g alcohol; one liter light beer to 22.0 g alcohol; one liter alcohol-free beer to 3.0 g alcohol; one liter of wine to 100.0 g alcohol; and one glass of spirits (0.02L) to 6.2 g alcohol. Grams per day were calculated as: (alcoholweekend + 5 * alcoholworking day)/7.

Acknowledgment

The KORA study was initiated and financed by the Helmholtz Zentrum München – German Research Center for Environmental Health, which is funded by the German Federal Ministry of Education and Research (BMBF) and by the State of Bavaria. Furthermore, KORA research was supported within the Munich Center of Health Sciences (MC-Health), Ludwig-Maximilians-Universität, as part of LMUinnovativ. The project was supported by the German Federal Ministry of Education and Research (BMBF) within the framework of the EU Joint Programming Initiative ‘A Healthy Diet for a Healthy Life’ (DIMENSION grant number 01EA1902A).

The study of Health in Pomerania (SHIP)-Trend [12] is a longitudinal population-based cohort study in West Pomerania, a region in the northeast of Germany, assessing the prevalence and incidence of common population-relevant diseases and their risk factors. Baseline examinations for SHIP-Trend were carried out between 2008 and 2012, comprising 4420 participants aged 20 to 81 years. DNA was extracted from blood samples of N=495 SHIP-Trend participants to assess DNA methylation using the Illumina HumanMethylationEPIC BeadChip array. Samples were randomly

selected based on availability of multiple OMICS data, excluding type II diabetes, and enriched for prevalent MI. The samples were taken between 07:00 AM and 04:00 PM, and serum aliquots were prepared for immediate analysis and for storage at -80 °C in the Integrated Research Biobank (Liconic, Liechtenstein). Processing of the DNA samples was performed at the Helmholtz Zentrum München. In the current study, 433 SHIP-Trend participants were available with data on alcohol intake and DNA methylation levels on the 144 predictive CpGs. Information regarding alcohol consumption was obtained by questionnaire: drink-specific quantity-frequency 30d [13].

Acknowledgment

SHIP is part of the Community Medicine Research net of the University of Greifswald, Germany, which is funded by the Federal Ministry of Education and Research (grants no. 01ZZ9603, 01ZZ0103, and 01ZZ0403), the Ministry of Cultural Affairs as well as the Social Ministry of the Federal State of Mecklenburg-West Pomerania, and the network 'Greifswald Approach to Individualized Medicine (GANI_MED)' funded by the Federal Ministry of Education and Research (grant 03IS2061A). DNA methylation data have been supported by the DZHK (grant 81X3400104). The University of Greifswald is a member of the Caché Campus program of the InterSystems GmbH.

The TwinsUK cohort was established in 1992 to recruit monozygotic and dizygotic same-sex twins [14], with over 14,000 twin participants (age range from 16 to 98 years old) from across the United Kingdom. In the current study, 713 female twin participants were included with data on alcohol intake and DNA methylation levels at the 144 CpGs. Alcohol consumption intakes in this sample of 713 twin participants was collected using self-reported questionnaires, as previously described [15]. Briefly, participants reported their average weekly alcohol intake, which was summarized as units per week and converted to grams/day (one unit of alcohol in the UK is defined as 7.9 grams [16]). DNA methylation data in the set of 713 TwinsUK participants was processed using BMIQ [17], as previously described [15]. A subset of 442 participants, hereby named TwinsUK2, further completed 131-item food frequency questionnaires [18]. TwinsUK2 habitual consumption of alcoholic beverages was converted to alcohol intake in grams/day. TwinsUK2 DNA methylation data was processed using the ENmix package [19]. In both TwinsUK datasets, alcohol intakes were obtained approximately

within two years of blood sample collection for DNA methylation profiling. Due to family relatedness epigenetic analyses of alcohol intake in TwisnUK datasets were carried out using linear mixed-effects models, fitting random effects for family and zygosity.

Acknowledgment

The project received support from the JPI ERA-HDHL DIMENSION project (BBSRC BB/S020845/1 to J.T.B.). TwinsUK is funded by the Wellcome Trust, Medical Research Council, European Union, Chronic Disease Research Foundation (CDRF), Zoe Global Ltd and the National Institute for Health Research (NIHR)-funded BioResource, Clinical Research Facility and Biomedical Research Centre based at Guy’s and St Thomas’ NHS Foundation Trust in partnership with King’s College London.

Microarray data acquisition and processing DNA methylation data

**Dataset**	**Methylation array**	**Tissue**	**Unrelated or Family**	**Normalization**
BIOS consortium	Illumina 450K array	Whole blood	Unrelated	DASEN
RS-III-1	Illumina 450K array	Whole blood	Unrelated	DASEN
KORA F4	Illumina 450K array	Whole blood	Unrelated	CPACOR
SHIP-Trend	Illumina EPIC array	Whole blood	Unrelated	CPACOR
TwinsUK	Illumina 450K array	Whole blood	Family	BMIQ
TwinsUK2	Illumina 450K array	Whole blood	Family	ENmix

BIOS consortium dataset includes participants from the Rotterdam Study, Cohort on Diabetes and Atherosclerosis Maastricht, the Netherlands Twin Register, Leiden Longevity Study, and the Prospective ALS Study Netherlands. BMIQ- beta-mixture quantile normalization method; CPACOR- Incorporating Control Probe Adjustment and reduction of global CORrelation; DASEN, a data-driven approach to preprocessing Illumina 450K; RS- The Rotterdam Study; SHIP- Study of Health in Pomerania-Trend cohort; TwinsUK- The TwinsUK Study; TwinsUK2- Subset of the TwinsUK Study.

References

1. Bonder MJ, Luijk R, Zhernakova DV, Moed M, Deelen P, Vermaat M, et al. Disease variants alter transcription factor levels and methylation of their binding sites. Nat Genet. 2017;49(1):131-8.
2. Ikram MA, Brusselle G, Ghanbari M, Goedegebure A, Ikram MK, Kavousi M, et al. Objectives, design and main findings until 2020 from the Rotterdam Study. Eur J Epidemiol. 2020;35(5):483-517.
3. van Greevenbroek MM, Jacobs M, van der Kallen CJ, Vermeulen VM, Jansen EH, Schalkwijk CG, et al. The cross-sectional association between insulin resistance and circulating complement C3 is partly explained by plasma alanine aminotransferase, independent of central obesity and general inflammation (the CODAM study). Eur J Clin Invest. 2011;41(4):372-9.
4. van Dam RM, Boer JM, Feskens EJ, Seidell JC. Parental history of diabetes modifies the association between abdominal adiposity and hyperglycemia. Diabetes Care. 2001;24(8):1454-9.
5. Willemsen G, Vink JM, Abdellaoui A, den Braber A, van Beek JH, Draisma HH, et al. The Adult Netherlands Twin Register: twenty-five years of survey and biological data collection. Twin Res Hum Genet. 2013;16(1):271-81.
6. Ligthart L, van Beijsterveldt CEM, Kevenaar ST, de Zeeuw E, van Bergen E, Bruins S, et al. The Netherlands Twin Register: Longitudinal Research Based on Twin and Twin-Family Designs. Twin Res Hum Genet. 2019;22(6):623-36.
7. Schoenmaker M, de Craen AJ, de Meijer PH, Beekman M, Blauw GJ, Slagboom PE, et al. Evidence of genetic enrichment for exceptional survival using a family approach: the Leiden Longevity Study. Eur J Hum Genet. 2006;14(1):79-84.
8. Westendorp RG, van Heemst D, Rozing MP, Frölich M, Mooijaart SP, Blauw GJ, et al. Nonagenarian siblings and their offspring display lower risk of mortality and morbidity than sporadic nonagenarians: The Leiden Longevity Study. J Am Geriatr Soc. 2009;57(9):1634-7.
9. Streppel MT, de Vries JH, Meijboom S, Beekman M, de Craen AJ, Slagboom PE, et al. Relative validity of the food frequency questionnaire used to assess dietary intake in the Leiden Longevity Study. Nutr J. 2013;12:75.
10. Huisman MH, de Jong SW, van Doormaal PT, Weinreich SS, Schelhaas HJ, van der Kooi AJ, et al. Population based epidemiology of amyotrophic lateral sclerosis using capture-recapture methodology. J Neurol Neurosurg Psychiatry. 2011;82(10):1165-70.
11. Holle R, Happich M, Lowel H, Wichmann HE, Group MKS. KORA--a research platform for population based health research. Gesundheitswesen. 2005;67 Suppl 1:S19-25.
12. Völzke H, Alte D, Schmidt CO, Radke D, Lorbeer R, Friedrich N, et al. Cohort profile: the study of health in Pomerania. Int J Epidemiol. 2011;40(2):294-307.
13. Baumeister SE, Schumann A, Nakazono TT, Alte D, Friedrich N, John U, et al. Alcohol consumption and out-patient services utilization by abstainers and drinkers. Addiction. 2006;101(9):1285-91.
14. Moayyeri A, Hammond CJ, Valdes AM, Spector TD. Cohort Profile: TwinsUK and healthy ageing twin study. Int J Epidemiol. 2013;42(1):76-85.
15. Liu C, Marioni RE, Hedman AK, Pfeiffer L, Tsai PC, Reynolds LM, et al. A DNA methylation biomarker of alcohol consumption. Mol Psychiatry. 2018;23(2):422-33.
16. Brennan A, Meng Y, Holmes J, Hill-McManus D, Meier PS. Potential benefits of minimum unit pricing for alcohol versus a ban on below cost selling in England 2014: modelling study. Bmj. 2014;349:g5452.
17. Teschendorff AE, Marabita F, Lechner M, Bartlett T, Tegner J, Gomez-Cabrero D, et al. A beta-mixture quantile normalization method for correcting probe design bias in Illumina Infinium 450 k DNA methylation data. Bioinformatics. 2013;29(2):189-96.
18. Teucher B, Skinner J, Skidmore PM, Cassidy A, Fairweather-Tait SJ, Hooper L, et al. Dietary patterns and heritability of food choice in a UK female twin cohort. Twin Res Hum Genet. 2007;10(5):734-48.
19. Xu Z, Niu L, Li L, Taylor JA. ENmix: a novel background correction method for Illumina HumanMethylation450 BeadChip. Nucleic Acids Res. 2016;44(3):e20.
