# Supplementary material for: Validating biomarkers and models for epigenetic inference of alcohol consumption from blood
Source: Clin Epigenetics. 2021 Oct 26;13:198. doi: 10.1186/s13148-021-01186-3 (PMC8549335; doi:10.1186/s13148-021-01186-3)

Additional file 4: Supplementary Figures

Figure S1. Accuracy of epigenetic inference of heavy drinkers *vs.* light and non-drinkers using different marker sets.

Figure S2. Accuracy of epigenetic inference of heavy drinkers *vs.*at-risk drinkers using different marker sets.

Figure S3. Accuracy of epigenetic inference of at-risk drinkers *vs.* non-drinkers using different marker sets.

Figure S4. Accuracy of epigenetic inference of at-risk drinkers *vs.* light drinkers using different marker sets.

Figure S5. Accuracy of epigenetic inference of light drinkers *vs.* non-drinkers using different marker sets.

Figure legend: Figures S1- S5

Prediction accuracy for alcohol consumption expressed as Area Under the Curve (AUC) using the CpG marker sets from Liu *et al.* 'Internal Validation': Mean AUC and SD from internal validation using ten-fold cross-validation in our model building dataset (6 cohorts, N= 2883). 'External Validation': AUCs from external validation by applying our model trained in the model building dataset to data from three external validation cohorts (Rotterdam Study, N= 648; SHIP-Trend, N= 433; and TwinsUK, N= 713 and N=442). Based on interview or self-reported information, non-drinkers were defined as participants with no alcohol consumption; light drinkers with an alcohol consumption of 0< g per day ⩽28 in men and 0< g per day ⩽14 in women; and heavy drinkers with an alcohol consumption of ⩾42 g per day in men and ⩾28 g per day in women.

Abbreviations: RS- The Rotterdam Study; TwinsUK- The TwinsUK Study; TwinsUK2- Subset of the TwinsUK Study; SHIP- Study of Health in Pomerania-Trend cohort

Figure S1. Accuracy of epigenetic inference of heavy drinkers *vs.* light and non-drinkers using different marker sets.

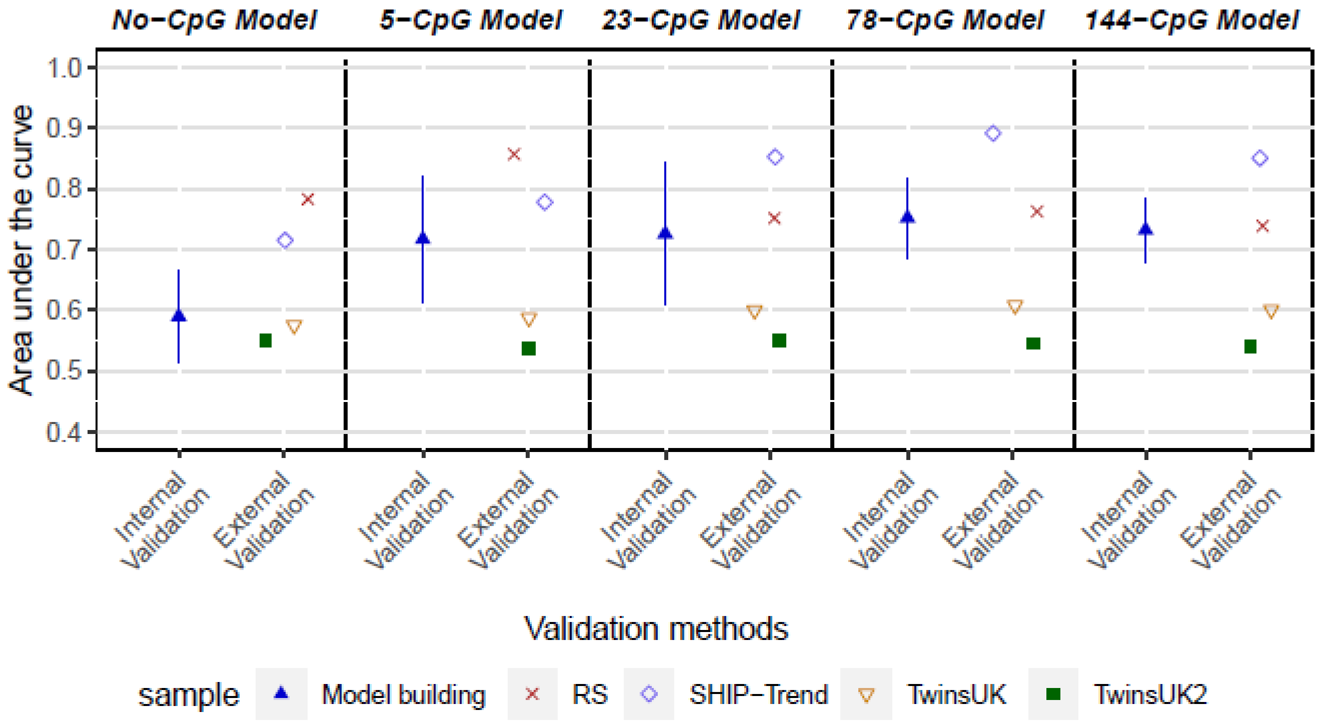

Figure S2. Accuracy of epigenetic inference of heavy drinkers *vs.* at-risk drinkers using different marker sets.

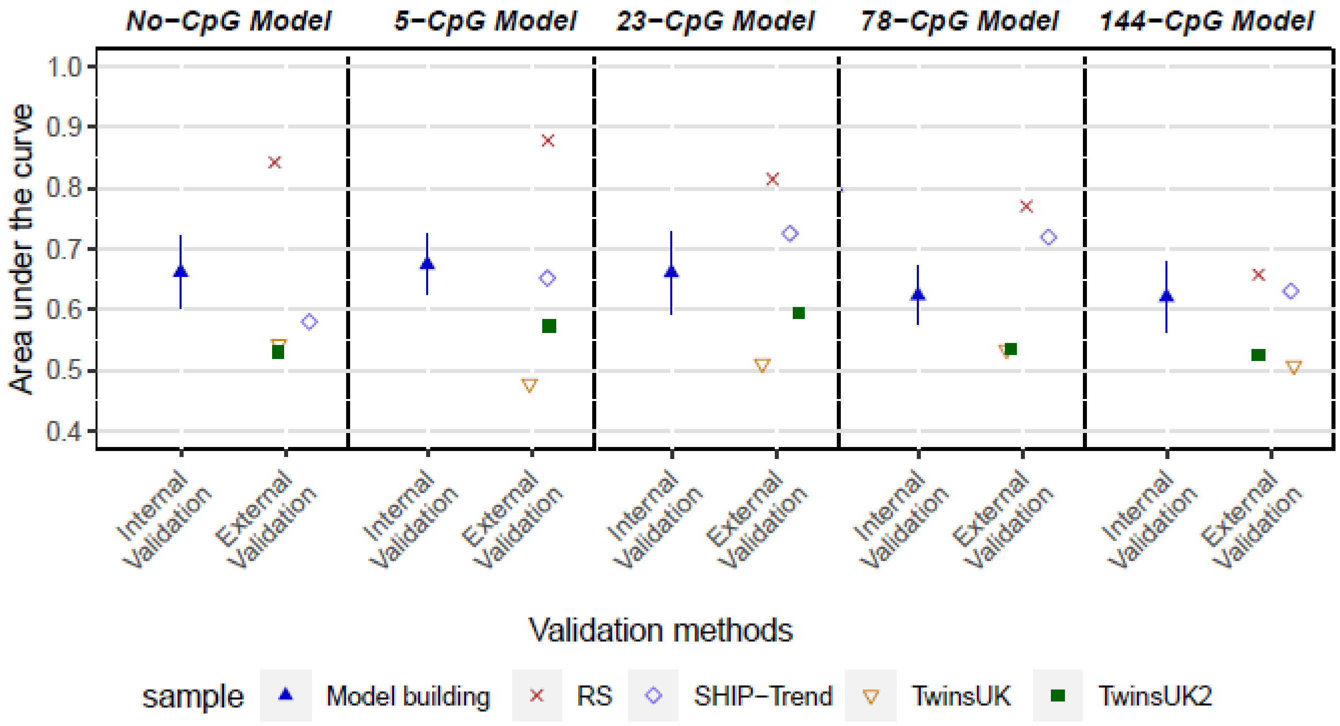

Figure S3. Accuracy of epigenetic inference of at-risk drinkers *vs.* non-drinkers using different marker sets.

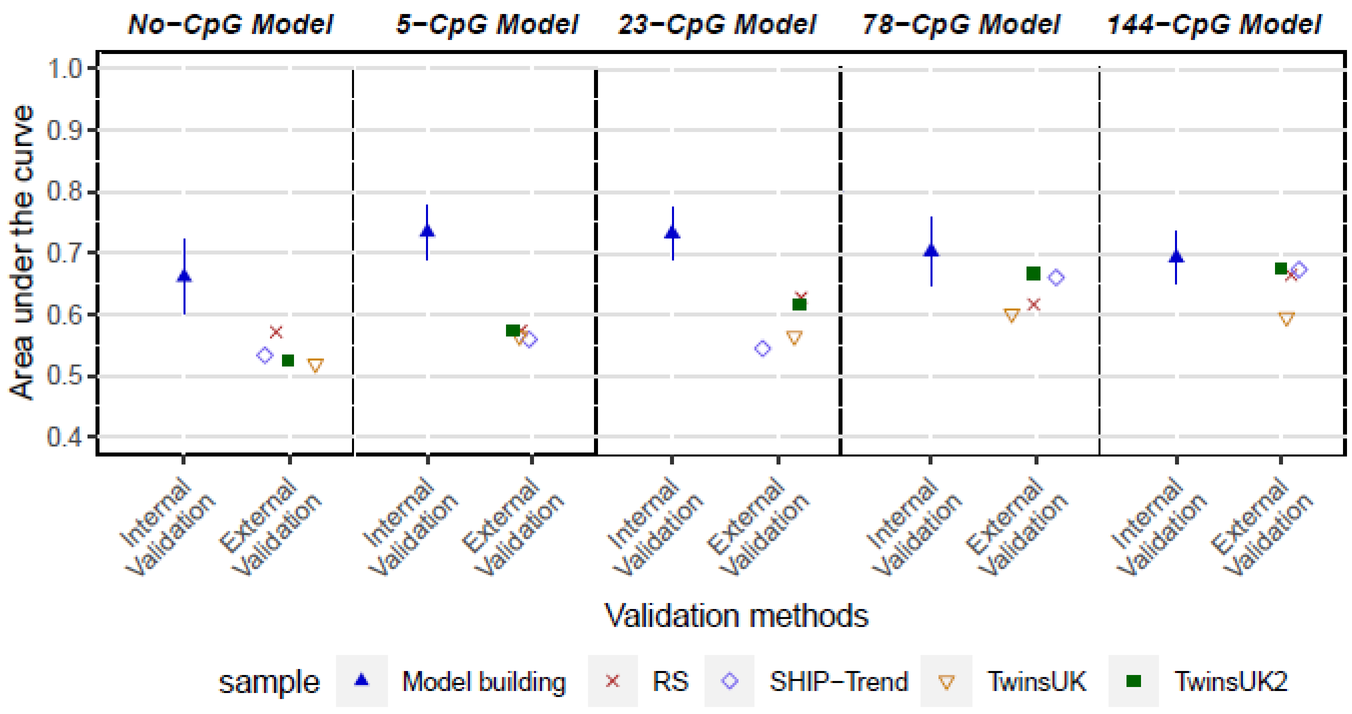

Figure S4. Accuracy of epigenetic inference of at-risk drinkers *vs.* light drinkers using different marker sets.

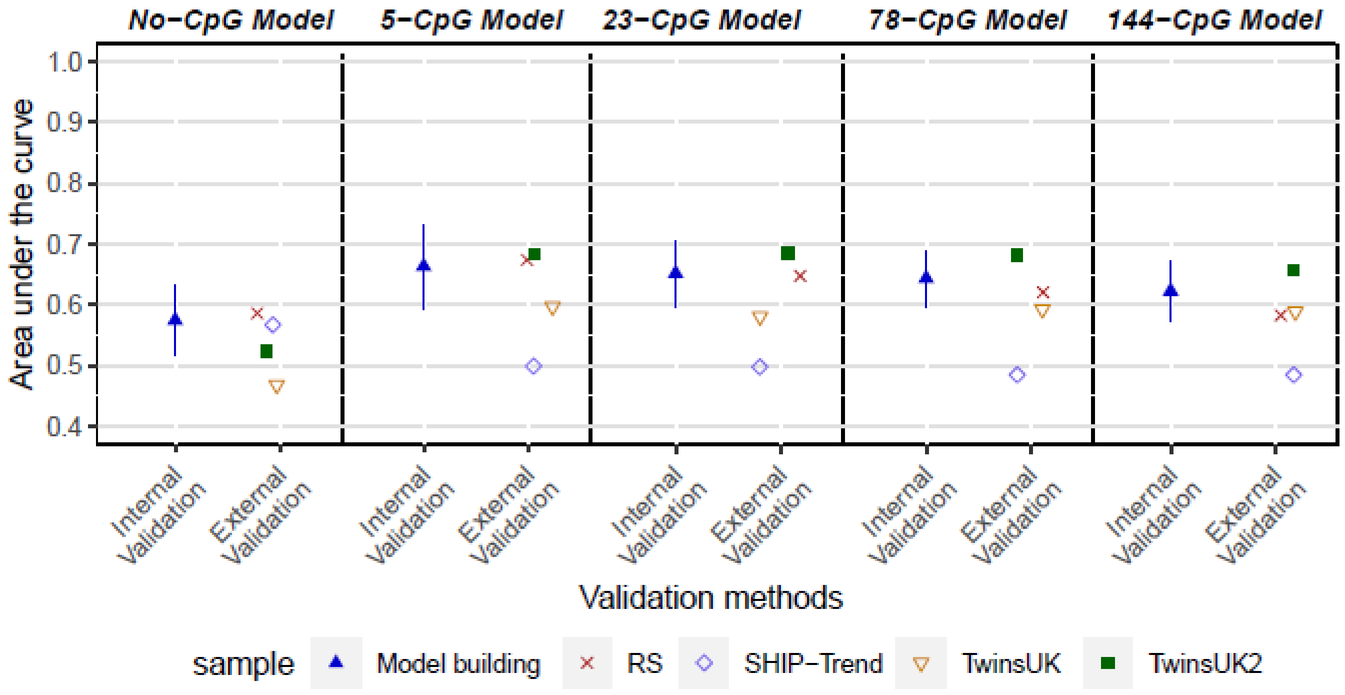

Figure S5. Accuracy of epigenetic inference of light drinkers *vs.* non-drinkers using different marker sets.

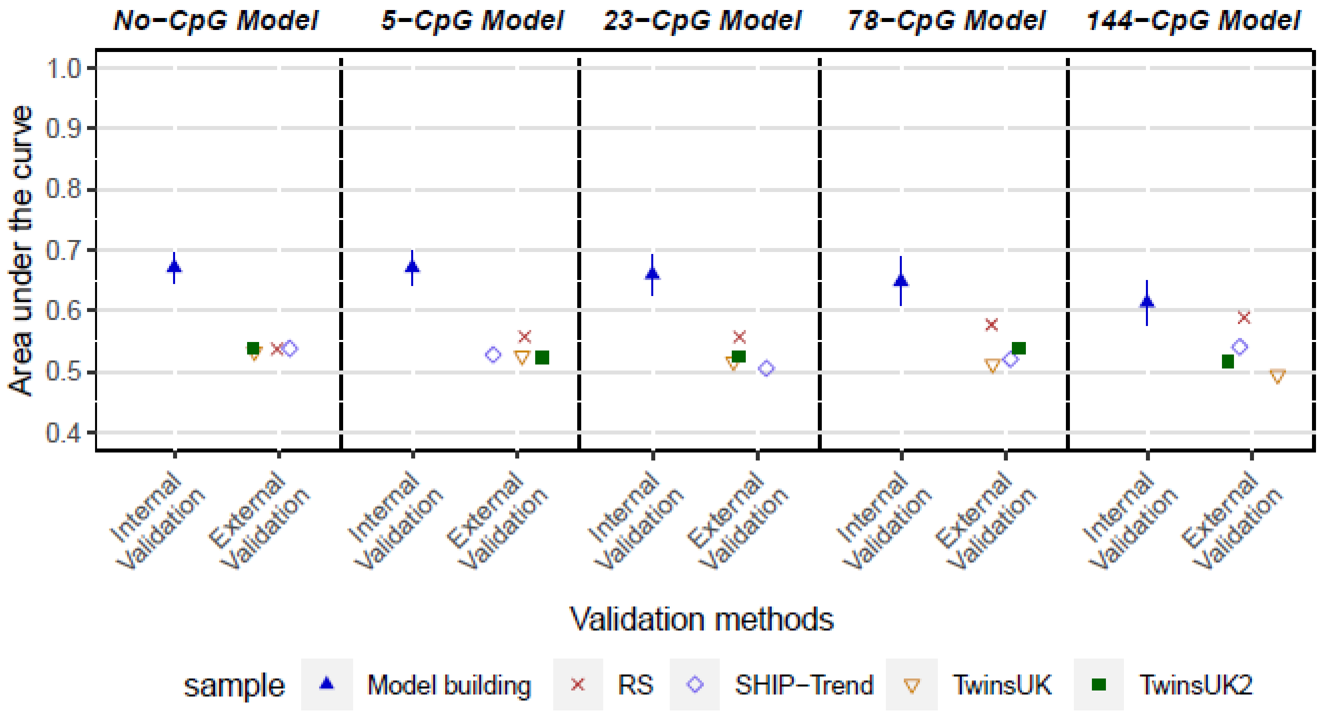

Supplement: Supplementary file 4 — Additional file 4: Figures S1–S5. Figure S1 shows the results for heavy drinkers vs. light and non-drinkers; Figure S2 shows the results for heavy drinkers vs. at-risk drinkers; Figure S3 shows the results for at-risk drinkers vs. non-drinkers; Figure S4 shows the results for at-risk drinkers vs. light drinkers; and Figure S5 shows the results for light drinkers vs. non-drinkers. [file 13148_2021_1186_MOESM4_ESM.pdf]
