# Supplementary material for: Validating biomarkers and models for epigenetic inference of alcohol consumption from blood
Source: Clin Epigenetics. 2021 Oct 26;13:198. doi: 10.1186/s13148-021-01186-3 (PMC8549335; doi:10.1186/s13148-021-01186-3)
Supplement: Supplementary file 5 — Additional file 5: Tables S3–S11. Table S3 shows the results for heavy drinkers vs. non-drinkers; Table S4 shows the results for heavy drinkers vs. light drinkers; Table S5 shows the results for heavy drinkers vs. light and non-drinkers; Table S6 shows the results for heavy drinkers vs. at-risk drinkers; Table S7 shows the results for at-risk drinkers vs. non-drinkers; Table S8 shows the results for at-risk drinkers vs. light drinkers; Table S9 shows the results for light drinkers vs. non-drinkers; Table S10 shows the results for heavy and at-risk drinkers vs. light and non-drinkers; and Table S11 shows the results for heavy, at-risk and light drinkers vs. non-drinker. [file 13148_2021_1186_MOESM5_ESM.pdf]

Additional file 5: Supplementary Tables

Table S3 Accuracy of epigenetic inference of heavy drinkers *vs.* non-drinkers using different marker sets

Table S4 Accuracy of epigenetic inference of heavy drinkers *vs.* light drinkers using different marker sets.

Table S5 Accuracy of epigenetic inference of heavy drinkers *vs.* light and non-drinkers using different marker sets.

Table S6 Accuracy of epigenetic inference of heavy drinkers *vs.* at-risk drinkers using different marker sets.

Table S7 Accuracy of epigenetic inference of at-risk drinkers *vs.* non-drinkers using different marker sets.

Table S8 Accuracy of epigenetic inference of at-risk drinkers *vs.* light drinkers using different marker sets.

Table S9 Accuracy of epigenetic inference of light drinkers *vs.* non-drinkers using different marker sets.

Table S10 Accuracy of epigenetic inference of heavy and at-risk drinkers *vs.* light and non-drinkers using different marker sets.

Table S11 Accuracy of epigenetic inference of heavy, at-risk and light drinkers *vs.* non-drinker using different marker sets.

Table legend: Table S3- Table S11

Prediction accuracy for alcohol consumption expressed as Area Under the Curve (AUC) using the CpG marker sets from Liu *et al.* 'Internal Validation': Obtained AUCs using ten-fold cross-validation in the model building data set; 'External Validation': AUCs from external validation by applying our model trained in the model building dataset to data from three external validation cohorts (Rotterdam Study, N= 648; SHIP-Trend, N= 433; and TwinsUK, N= 713 and N=442). Based on interview or self-reported information, non-drinkers were defined as participants with no alcohol consumption; light drinkers with an alcohol consumption of 0< g per day ⩽28 in men and 0< g per day ⩽14 in women; and heavy drinkers with an alcohol consumption of ⩾42 g per day in men and ⩾28 g per day in women. Abbreviations: RS- The Rotterdam Study; TwinsUK- The TwinsUK Study; TwinsUK2- Subset of the TwinsUK Study; SHIP- Study of Health in Pomerania-Trend cohort; ABS- Null model including only age, body mass index, and sex.

Table S3 Accuracy of epigenetic inference of heavy drinkers *vs.* non-drinkers using different marker sets.

Marker set	*Internal validation*	*External validation*			
		RS	*SHIP-Trend*	*TwinsUK*	*TwinsUK2*
144-CpGs	0.78±0.06	0.80	0.84	0.68	0.60
78-CpGs	0.81±0.06	0.85	0.83	0.66	0.63
23-CpGs	0.83±0.05	0.81	0.87	0.65	0.61
5-CpGs	0.83±0.05	0.89	0.79	0.60	0.58
ABS	0.73±0.05	0.81	0.68	0.52	0.50

Table S4 Accuracy of epigenetic inference of heavy drinkers *vs.* light drinkers using different marker sets.

Marker set	*Internal validation*	*External validation*			
		RS	*SHIP-Trend*	*TwinsUK*	*TwinsUK2*
144-CpGs	0.73±0.06	0.72	0.84	0.57	0.53
78-CpGs	0.74±0.04	0.76	0.89	0.57	0.53
23-CpGs	0.74±0.04	0.73	0.85	0.57	0.54
5-CpGs	0.72±0.04	0.84	0.77	0.56	0.52
ABS	0.59±0.07	0.76	0.71	0.57	0.55

Table S5 Accuracy of epigenetic inference of heavy drinkers *vs.* light and non-drinkers using different marker sets.

Marker set	*Internal validation*	*External validation*			
		RS	*SHIP-Trend*	*TwinsUK*	*TwinsUK2*
144-CpGs	0.73±0.05	0.74	0.85	0.60	0.54
78-CpGs	0.75±0.07	0.76	0.89	0.61	0.55
23-CpGs	0.73±0.12	0.75	0.85	0.60	0.55
5-CpGs	0.72±0.10	0.86	0.78	0.59	0.54
ABS	0.59±0.08	0.78	0.72	0.58	0.55

Table S6 Accuracy of epigenetic inference of heavy drinkers *vs.* at-risk drinkers using different marker sets.

Marker set	*Internal validation*	*External validation*			
		RS	*SHIP-Trend*	*TwinsUK*	*TwinsUK2*
144-CpGs	0.62±0.06	0.66	0.63	0.51	0.53
78-CpGs	0.62±0.05	0.77	0.72	0.53	0.54
23-CpGs	0.66±0.07	0.82	0.73	0.51	0.60
5-CpGs	0.67±0.05	0.88	0.65	0.48	0.57
ABS	0.66±0.06	0.84	0.58	0.54	0.53

Table S7 Accuracy of epigenetic inference of at-risk drinkers *vs.* non-drinkers using different marker sets.

	Internal	*External validation*			
Marker set	*validation*	*RS*	*SHIP-Trend*	*TwinsUK*	*TwinsUK2*
144-CpGs	0.69±0.04	0.67	0.67	0.60	0.68
78-CpGs	0.70±0.06	0.62	0.66	0.60	0.67
23-CpGs	0.73±0.04	0.63	0.55	0.57	0.62
5-CpGs	0.73±0.04	0.57	0.56	0.56	0.57
ABS	0.66±0.06	0.57	0.53	0.52	0.53

Table S8 Accuracy of epigenetic inference of at-risk drinkers *vs.* light drinkers using different marker sets.

Marker set	*Internal validation*	*External validation*			
		RS	*SHIP-Trend*	*TwinsUK*	*TwinsUK2*
144-CpGs	0.62±0.05	0.58	0.49	0.59	0.66
78-CpGs	0.64±0.05	0.62	0.49	0.59	0.68
23-CpGs	0.65±0.05	0.65	0.50	0.58	0.69
5-CpGs	0.66±0.07	0.67	0.50	0.60	0.68
ABS	0.58±0.06	0.59	0.57	0.47	0.52

Table S9 Accuracy of epigenetic inference of light drinkers *vs.* non-drinkers using different marker sets.

Marker set	*Internal validation*	*External validation*			
		RS	*SHIP-Trend*	*TwinsUK*	*TwinsUK2*
144-CpGs	0.61±0.04	0.59	0.54	0.50	0.52
78-CpGs	0.65±0.04	0.58	0.52	0.51	0.54
23-CpGs	0.66±0.03	0.56	0.51	0.52	0.53
5-CpGs	0.67±0.03	0.56	0.53	0.53	0.52
ABS	0.67±0.03	0.54	0.54	0.53	0.54

Table S10 Accuracy of epigenetic inference of heavy and at-risk drinkers *vs.* light and non-drinkers using different marker sets.

Marker set	*Internal validation*	*External validation*			
		RS	*SHIP-Trend*	*TwinsUK*	*TwinsUK2*
144-CpGs	0.67±0.02	0.61	0.66	0.61	0.61
78-CpGs	0.68±0.03	0.65	0.70	0.60	0.65
23-CpGs	0.68±0.04	0.65	0.66	0.60	0.63
5-CpGs	0.67±0.05	0.65	0.66	0.60	0.62

Table S11 Accuracy of epigenetic inference of heavy, at-risk and light drinkers *vs.* non-drinker using different marker sets.

	Internal	*External validation*			
Marker set	*validation*	*RS*	*SHIP-Trend*	*TwinsUK*	*TwinsUK2*
144-CpGs	0.55±0.04	0.61	0.62	0.57	0.59
78-CpGs	0.56±0.05	0.61	0.60	0.59	0.63
23-CpGs	0.55±0.05	0.58	0.60	0.58	0.57
5-CpGs	0.54±0.04	0.61	0.54	0.58	0.59
