# Supplementary material for: Validating biomarkers and models for epigenetic inference of alcohol consumption from blood
Source: Clin Epigenetics. 2021 Oct 26;13:198. doi: 10.1186/s13148-021-01186-3 (PMC8549335; doi:10.1186/s13148-021-01186-3)
Supplement: Supplementary file 6 — Additional file 6. We provide the data, methods, and results regarding the replication of the Liu et al. approach; including accompanying supplementary Tables S12–S20, and supplementary Figures S6–S14. Table S12 shows the results for heavy drinkers vs. non-drinkers; Table S13 shows the results for heavy drinkers vs. light drinkers; Table S14 shows the results for heavy drinkers vs. light and non-drinkers; Table S15 shows the results for heavy drinkers vs. at-risk drinkers; Table S16 shows the results for at-risk drinkers vs. non-drinkers; Table S17 shows the results for at-risk drinkers vs. light drinkers; Table S18 shows the results for light drinkers vs. non-drinkers; Table S19 shows the results for heavy and at-risk drinkers vs. light and non-drinkers; and Table S20 shows the results for heavy, at-risk and light drinkers vs. non-drinker. Figure S6 shows the results for heavy drinkers vs. non-drinkers; Figure S7 shows the results for heavy drinkers vs. light drinkers; Figure S8 shows the results for heavy drinkers vs. light and non-drinkers; Figure S9 shows the results for heavy drinkers vs. at-risk drinkers; Figure S10 shows the results for at-risk drinkers vs. non-drinkers; Figure S11 shows the results for at-risk drinkers vs. light drinkers; Figure S12 shows the results for light drinkers vs. non-drinkers; Figure S13 shows the results for heavy and at-risk drinkers vs. light and non-drinkers; and Figure S14 shows the results for heavy, at-risk and light drinkers vs. non-drinker. [file 13148_2021_1186_MOESM6_ESM.pdf]

Additional file 6: Replication Liu *et al.* method.

Content

Replication Liu *et al.* method: Supplementary Methods 2
Replication Liu *et al.* method: Supplementary Results 3
Replication Liu *et al.* method: Supplementary Tables S12-S20 4
Replication Liu *et al.* method: Supplementary Figures S6-S14 16

Replication Liu *et al.* method: Supplementary Methods

We replicated the methodological approach previously used by Liu *et al.* [1] to validate the proposed prediction models and biomarker sets in our model building dataset comprising the combined data from the BIOS consortium and KORA (N=2883) [2-8]. Hence, the prediction models were trained using binomial regression analysis with the alcohol categories (coded as 1/0) as the dependent variable and age, sex, and BMI without (the null model) or with a set of (the residuals of the) CpGs as the independent variables (Alcohol category = age + sex + BMI (+ResCpGs$_{5, 23, 78, 144}$)). For this purpose, the "glm" function with "binomial" as family and "logit" as link were used. The models were then applied to the same participants using the "predict" function. The prediction performance of the models was assessed using "roc" (R-package "pROC") that calculates the AUC per model. For this, we tested the models in the same dataset as applied for model building, as previously done by Liu *et al.* and thus, we did here for reasons of outcome compatibility.

Replication Liu *et al.* method: Supplementary Results

The AUCs for the seven prediction models obtained for the five datasets used by Liu *et al.* [1] are denoted as 'Discovery' in **Table S12 – S18** and **Figure S6 – S14** (see below). To make the newly obtained prediction outcomes directly comparable with those reported by Liu *et al*., we implemented the same prediction approach previously used by Liu *et al.* in our independent data, i.e., i) the same CpGs, ii) the same method of training and testing the models in the same dataset, and iii) only using a subset of categories and therefore participants in the prediction models. To this end, we used the complete model building dataset (N=2883), which has no overlap in participants with the original prediction marker discovery EWAS conducted by Liu *et al*. [1]. We found that the obtained AUCs, denoted as 'Replication' in **Table S12 – S18** and **Figure S6 – S14**, were similarly high compared to those reported by Liu *et al.* for all four CpG marker sets and all prediction models. Also, in line with Liu *et al.*, we observed an AUC increase by including CpGs in the models (Alcohol category = age + sex + BMI + ResCpGs) relative to the null models without CpGs (Alcohol category = age + sex + BMI), as well as a gradual AUC rise with an increased number of CpGs in the models from 5 CpGs, to 23, 78, and the full model with 144 CpGs.

Further, as reported by Liu *et al.*, we observed that the most accurate prediction was obtained with the full 144-CpG model for the two extreme categories heavy *vs.* non-drinkers, with an AUC of 0.95 in our model building dataset, which was in the range of 0.91-1.0 reported by Liu *et al.* (**Table S12** and **Figure S6**). For heavy *vs.* light drinkers, the 144-CpG model delivered an AUC of 0.86 in our model building dataset, which agrees with the range of 0.86-1.00 reported by Liu *et al.* (**Table S13** and **Figure S7**). Strongly similar results obtained from our model building dataset compared to those reported by Liu *et al.* were also seen for all other models.

Thus, applying the same biomarkers, models, and methodology previously used by Liu *et al.* in our independent dataset successfully replicated the high accuracies in epigenetic inference of alcohol intake from blood as reported by Liu *et al*.

Replication Liu *et al.* method: Supplementary Tables S12-S20

Table S12 Accuracy of epigenetic inference of heavy drinkers *vs.* non-drinkers using different marker sets

Table S13 Accuracy of epigenetic inference of heavy drinkers *vs.* light drinkers using different marker sets.

Table S14 Accuracy of epigenetic inference of heavy drinkers *vs.* light and non-drinkers using different marker sets.

Table S15 Accuracy of epigenetic inference of heavy drinkers *vs.* at-risk drinkers using different marker sets.

Table S16 Accuracy of epigenetic inference of at-risk drinkers *vs.* non-drinkers using different marker sets.

Table S17 Accuracy of epigenetic inference of at-risk drinkers *vs.* light drinkers using different marker sets.

Table S18 Accuracy of epigenetic inference of light drinkers *vs.* non-drinkers using different marker sets.

Table S19 Accuracy of epigenetic inference of heavy and at-risk drinkers *vs.* light and non-drinkers using different marker sets.

Table S20 Accuracy of epigenetic inference of heavy, at-risk and light drinkers *vs.* non-drinker using different marker sets.

Table legend: Table S12- Table S18

Prediction accuracy for alcohol consumption expressed as Area Under the Curve (AUC) using the CpG marker sets from Liu *et al.* 'Discovery': AUCs per cohort from Liu *et al.* [1] as derived from the Liu *et al.* published figures. 'Replication': Obtained AUCs in our model building dataset by training and testing the model in the same dataset; 'Internal Validation': Obtained AUCs using ten-fold cross-validation in the model building data set; 'External Validation': AUCs from external validation by applying our model trained in the model building dataset to data from three external validation cohorts (Rotterdam Study, N= 648; SHIP-Trend, N= 433; and TwinsUK, N= 713 and N=442). Based on interview or self-reported information, non-drinkers were defined as participants with no alcohol consumption; light drinkers with an alcohol consumption of 0< g per day ⩽28 in men and 0< g per day ⩽14 in women; and heavy drinkers with an alcohol consumption of ⩾42 g per day in men and ⩾28 g per day in women. Abbreviations: ARIC- The Atherosclerosis Risk in Communities study; FHS- The Framingham Heart Study; KORA F4- The Cooperative Health Research in the Region of Augsburg study; LBC1936- The Lothian Birth Cohort 1936; MESA- The Multi-Ethnic Study of Atherosclerosis; RS- The Rotterdam Study; TwinsUK- The TwinsUK Study; TwinsUK2- Subset of the TwinsUK Study; SHIP- Study of Health in Pomerania-Trend cohort; ABS- Null model including only age, body mass index and sex.

Table legend: Table S19- Table S20

Prediction accuracy for alcohol consumption expressed as Area Under the Curve (AUC) using the CpG marker sets from Liu *et al.* for the new alcohol prediction model including all categories and therefore all participants. 'Replication': Obtained AUC in the model building data set by training and testing the model in the same dataset; 'Internal Validation': Obtained AUCs using ten-fold cross-validation in the model building data set; 'External Validation': AUCs obtained by training the models in our model building dataset and subsequently apply them to data from three external validation cohorts (Rotterdam Study, N= 648; SHIP-Trend, N= 433; and TwinsUK, N= 713 and N=442). Non-drinkers: participants with no alcohol consumption; light drinkers: participants with an alcohol consumption of 0< g per day ⩽28 in men and 0< g per day ⩽14 in women; heavy drinkers:

participants with an alcohol consumption of ⩾42 g per day in men and ⩾28 g per day in.

Abbreviations: RS- The Rotterdam Study; TwinsUK- The TwinsUK Study; TwinsUK2- Subset of the TwinsUK Study; SHIP- Study of Health in Pomerania-Trend cohort.

Table S12 Accuracy of epigenetic inference of heavy drinkers *vs.* non-drinkers using different marker sets.

	Discovery by Liu et al.						*Internal*	*External validation*			
Marker set	*ARIC*	*FHS*	*KORA F4*	*LBC 1936*	*MESA*	*Replication*	*validation*	*RS*	*SHIP-Trend*	*TwinsUK*	*TwinsUK2*
144-CpGs	0.91	0.99	0.96	1.0	1.0	0.95	0.78±0.06	0.80	0.84	0.68	0.60
78-CpGs	0.89	0.96	0.91	0.98	1.0	0.90	0.81±0.06	0.85	0.83	0.66	0.63
23-CpGs	0.85	0.91	0.86	0.91	0.91	0.86	0.83±0.05	0.81	0.87	0.65	0.61
5-CpGs	0.84	0.88	0.83	0.87	0.83	0.84	0.83±0.05	0.89	0.79	0.60	0.58
ABS	0.80	0.67	0.75	0.67	0.63	0.72	0.73±0.05	0.81	0.68	0.52	0.50

Table S13 Accuracy of epigenetic inference of heavy drinkers *vs.* light drinkers using different marker sets.

	Discovery by Liu et al.						*Internal*	*External validation*			
Marker set	*ARIC*	*FHS*	*KORA F4*	*LBC 1936*	*MESA*	*Replication*	*validation*	*RS*	*SHIP-Trend*	*TwinsUK*	*TwinsUK2*
144-CpGs	0.86	0.93	0.87	0.96	1.0	0.86	0.73±0.06	0.72	0.84	0.57	0.53
78-CpGs	0.82	0.90	0.82	0.90	0.96	0.82	0.74±0.04	0.76	0.89	0.57	0.53
23-CpGs	0.73	0.84	0.74	0.82	0.84	0.76	0.74±0.04	0.73	0.85	0.57	0.54
5-CpGs	0.68	0.81	0.72	0.77	0.81	0.73	0.72±0.04	0.84	0.77	0.56	0.52
ABS	0.59	0.58	0.61	0.54	0.59	0.60	0.59±0.07	0.76	0.71	0.57	0.55

Table S14 Accuracy of epigenetic inference of heavy drinkers *vs.* light and non-drinkers using different marker sets.

	Discovery by Liu et al.						*Internal*	*External validation*			
Marker set	*ARIC*	*FHS*	*KORA F4*	*LBC 1936*	*MESA*	*Replication*	*validation*	*RS*	*SHIP-Trend*	*TwinsUK*	*TwinsUK2*
144-CpGs	0.90	0.94	0.89	0.96	1.0	0.87	0.73±0.05	0.74	0.85	0.60	0.54
78-CpGs	0.88	0.91	0.85	0.90	0.96	0.83	0.75±0.07	0.76	0.89	0.61	0.55
23-CpGs	0.84	0.87	0.79	0.84	0.85	0.78	0.73±0.12	0.75	0.85	0.60	0.55
5-CpGs	0.83	0.84	0.76	0.79	0.83	0.75	0.72±0.10	0.86	0.78	0.59	0.54
ABS	0.79	0.61	0.66	0.56	0.61	0.61	0.59±0.08	0.78	0.72	0.58	0.55

Table S15 Accuracy of epigenetic inference of heavy drinkers *vs.* at-risk drinkers using different marker sets.

	Discovery by Liu et al.						*Internal*	*External validation*			
Marker set	*ARIC*	*FHS*	*KORA F4*	*LBC 1936*	*MESA*	*replication*	*validation*	*RS*	*SHIP-Trend*	*TwinsUK*	*TwinsUK2*
144-CpGs	1.0	0.89	0.91	1.0	1.0	0.88	0.62±0.06	0.66	0.63	0.51	0.53
78-CpGs	0.91	0.84	0.85	1.0	1.0	0.80	0.62±0.05	0.77	0.72	0.53	0.54
23-CpGs	0.80	0.73	0.76	0.82	0.87	0.73	0.66±0.07	0.82	0.73	0.51	0.60
5-CpGs	0.73	0.69	0.74	0.64	0.72	0.69	0.67±0.05	0.88	0.65	0.48	0.57
ABS	0.69	0.59	0.72	0.64	0.64	0.67	0.66±0.06	0.84	0.58	0.54	0.53

Table S16 Accuracy of epigenetic inference of at-risk drinkers *vs.* non-drinkers using different marker sets.

	Discovery by Liu et al.						*Internal*	*External validation*			
Marker set	*ARIC*	*FHS*	*KORA F4*	*LBC 1936*	*MESA*	*Replication*	*validation*	*RS*	*SHIP-Trend*	*TwinsUK*	*TwinsUK2*
144-CpGs	0.91	0.93	0.86	1.0	1.0	0.86	0.69±0.04	0.67	0.67	0.60	0.68
78-CpGs	0.86	0.88	0.81	0.93	1.0	0.81	0.70±0.06	0.62	0.66	0.60	0.67
23-CpGs	0.74	0.81	0.71	0.84	0.83	0.76	0.73±0.04	0.63	0.55	0.57	0.62
5-CpGs	0.71	0.77	0.68	0.77	0.75	0.74	0.73±0.04	0.57	0.56	0.56	0.57
ABS	0.68	0.63	0.59	0.61	0.69	0.66	0.66±0.06	0.57	0.53	0.52	0.53

Table S17 Accuracy of epigenetic inference of at-risk drinkers *vs.* light drinkers using different marker sets.

	Discovery by Liu et al.						*Internal*	*External validation*			
Marker set	*ARIC*	*FHS*	*KORA F4*	*LBC 1936*	*MESA*	*Replication*	*validation*	*RS*	*SHIP-Trend*	*TwinsUK*	*TwinsUK2*
144-CpGs	1.0	0.82	0.80	0.90	1.0	0.76	0.62±0.05	0.58	0.49	0.59	0.66
78-CpGs	1.0	0.77	0.76	0.82	0.94	0.73	0.64±0.05	0.62	0.49	0.59	0.68
23-CpGs	0.84	0.81	0.69	0.73	0.75	0.69	0.65±0.05	0.65	0.50	0.58	0.69
5-CpGs	0.74	0.70	0.65	0.67	0.67	0.67	0.66±0.07	0.67	0.50	0.60	0.68
ABS	0.70	0.59	0.61	0.61	0.65	0.58	0.58±0.06	0.59	0.57	0.47	0.52

Table S18 Accuracy of epigenetic inference of light drinkers *vs.* non-drinkers using different marker sets.

	Discovery by Liu et al.						*Internal*	*External validation*			
Marker set	*ARIC*	*FHS*	*KORA F4*	*LBC 1936*	*MESA*	*Replication*	*validation*	*RS*	*SHIP-Trend*	*TwinsUK*	*TwinsUK2*
144-CpGs	0.95	0.75	0.80	0.82	0.87	0.74	0.61±0.04	0.59	0.54	0.50	0.52
78-CpGs	0.88	0.71	0.76	0.76	0.76	0.72	0.65±0.04	0.58	0.52	0.51	0.54
23-CpGs	0.83	0.65	0.70	0.70	0.66	0.69	0.66±0.03	0.56	0.51	0.52	0.53
5-CpGs	0.80	0.64	0.68	0.68	0.60	0.68	0.67±0.03	0.56	0.53	0.53	0.52
ABS	0.78	0.61	0.66	0.64	0.57	0.67	0.67±0.03	0.54	0.54	0.53	0.54

Table S19 Accuracy of epigenetic inference of heavy and at-risk drinkers *vs.* light and non-drinkers using different marker sets.

Marker set	*Replication*	*Internal validation*	*External validation*			
			RS	*SHIP-Trend*	*TwinsUK*	*TwinsUK2*
144-CpGs	0.76	0.67±0.02	0.61	0.66	0.61	0.61
78-CpGs	0.74	0.68±0.03	0.65	0.70	0.60	0.65
23-CpGs	0.70	0.68±0.04	0.65	0.66	0.60	0.63
5-CpGs	0.67	0.67±0.05	0.65	0.66	0.60	0.62

Table S20 Accuracy of epigenetic inference of heavy, at-risk and light drinkers *vs.* non-drinker using different marker sets.

Marker set	*Replication*	*Internal validation*	*External validation*			
			RS	*SHIP-Trend*	*TwinsUK*	*TwinsUK2*
144-CpGs	0.69	0.55±0.04	0.61	0.62	0.57	0.59
78-CpGs	0.66	0.56±0.05	0.61	0.60	0.59	0.63
23-CpGs	0.59	0.55±0.05	0.58	0.60	0.58	0.57
5-CpGs	0.55	0.54±0.04	0.61	0.54	0.58	0.59

Replication Liu *et al.* method: Supplementary Figures S6-S14

Figure S6. Accuracy of epigenetic inference of heavy drinkers *vs.* non-drinkers using different marker sets.

Figure S7. Accuracy of epigenetic inference of heavy drinkers *vs.* light drinkers using different marker sets.

Figure S8. Accuracy of epigenetic inference of heavy drinkers *vs.* light and non-drinkers using different marker sets.

Figure S9. Accuracy of epigenetic inference of heavy drinkers *vs.*at-risk drinkers using different marker sets.

Figure S10. Accuracy of epigenetic inference of at-risk drinkers *vs.* non-drinkers using different marker sets.

Figure S11. Accuracy of epigenetic inference of at-risk drinkers *vs.* light drinkers using different marker sets.

Figure S12. Accuracy of epigenetic inference of light drinkers *vs.* non-drinkers using different marker sets.

Figure S13. Accuracy of epigenetic inference of heavy and at-risk drinkers *vs.* light and non-drinkers using different marker sets.

Figure S14. Accuracy of epigenetic inference of heavy, at-risk and light drinkers *vs.* non-drinkers using different marker sets.

Figure legend: Figures S6- S12

Prediction accuracy for alcohol consumption expressed as Area Under the Curve (AUC) using the CpG marker sets from Liu *et al.* [1]. 'Discovery': AUCs per cohort from Liu *et al.* as derived from the Liu *et al.* published figures. 'Replication': AUCs obtained in our model building dataset (6 cohorts, N= 2883) based on the Liu *et al*. prediction approach using the same data for model building and model testing. 'Internal Validation': Mean AUC and SD from internal validation using ten-fold cross-validation in our model building dataset. 'External Validation': AUCs from external validation by applying our model trained in the model building dataset to data from three external validation cohorts (Rotterdam Study, N= 648; SHIP-Trend, N= 433; and TwinsUK, N= 713 and

N=442). Based on interview or self-reported information, non-drinkers were defined as participants with no alcohol consumption; light drinkers with an alcohol consumption of 0< g per day ⩽28 in men and 0< g per day ⩽14 in women; and heavy drinkers with an alcohol consumption of ⩾42 g per day in men and ⩾28 g per day in women. Abbreviations: ARIC- The Atherosclerosis Risk in Communities study; FHS- The Framingham Heart Study; KORA F4- The Cooperative Health Research in the Region of Augsburg study; LBC1936- The Lothian Birth Cohort 1936; MESA- The Multi-Ethnic Study of Atherosclerosis; RS- The Rotterdam Study; TwinsUK- The TwinsUK Study; TwinsUK2- Subset of the TwinsUK Study; SHIP- Study of Health in Pomerania-Trend cohort

Figure legend: Figures S13-S14

Prediction accuracy for alcohol consumption expressed as Area Under the Curve (AUC) using the CpG marker sets from Liu *et al.* [1]. In these models, all available participants from all categories were included. 'Model Building': AUCs from our model building dataset (6 cohorts, N= 2883) based on the Liu *et al*. prediction approach using the same data for model building and model testing. 'Internal Validation': Mean AUC and SD from internal validation using ten-fold cross-validation in our model building data set. 'External Validation': AUCs from external validation by applying our model trained in the model building dataset to independent data from three external validation cohorts (Rotterdam Study, N= 648; SHIP-Trend, N= 433; and TwinsUK, N= 713 and N= 442). Based on interview or self-reported information, non-drinkers were defined as participants with no alcohol consumption; light drinkers with an alcohol consumption of 0< g per day ⩽28 in men and 0< g per day ⩽14 in women; and heavy drinkers with an alcohol consumption of ⩾42 g per day in men and ⩾28 g per day in women. Abbreviations: RS- The Rotterdam Study; TwinsUK- The TwinsUK Study; TwinsUK2- Subset of the TwinsUK Study; SHIP- Study of Health in Pomerania-Trend cohort.

Figure S6. Accuracy of epigenetic inference of heavy drinkers *vs.* non-drinkers using different marker sets.

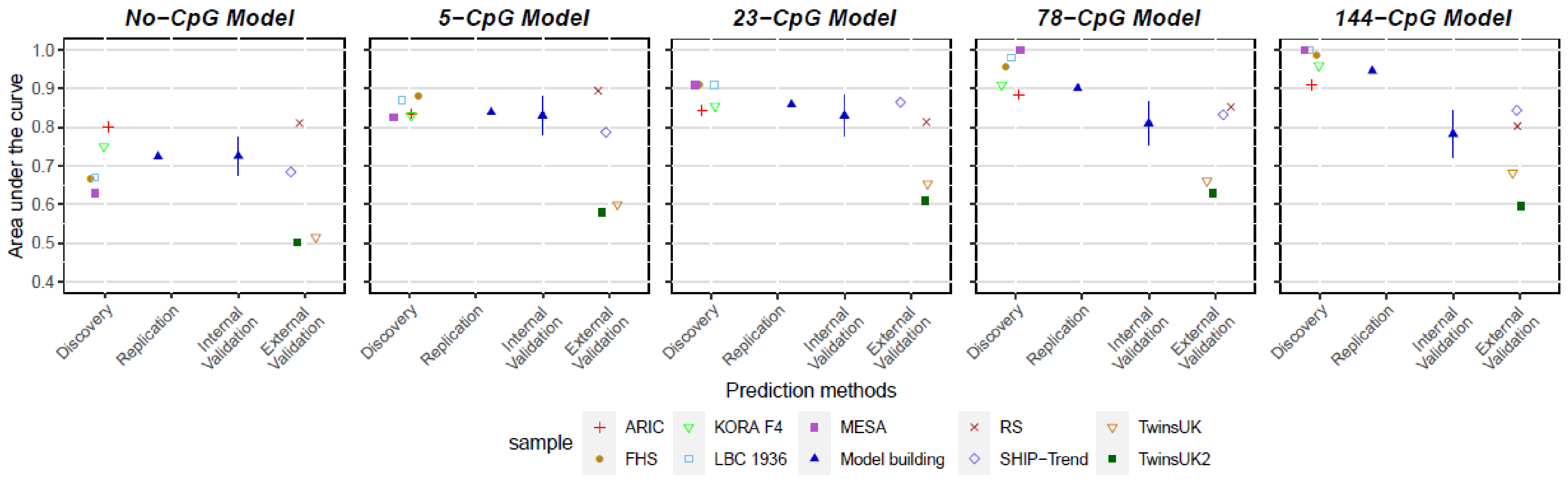

Figure S7. Accuracy of epigenetic inference of heavy drinkers *vs.* light drinkers using different marker sets.

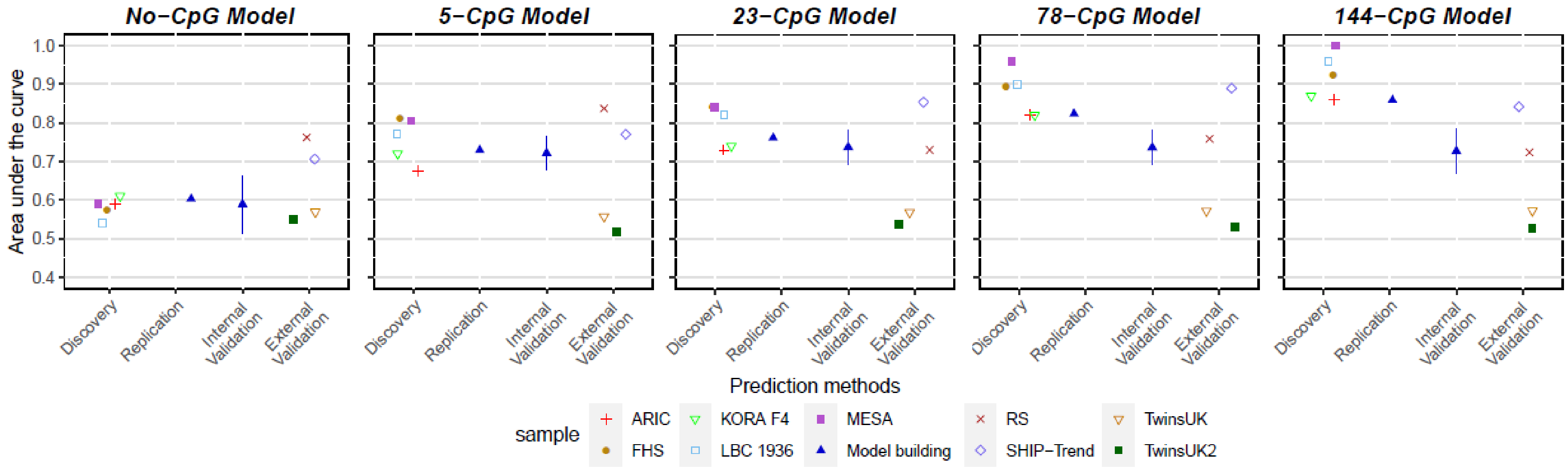

Figure S8. Accuracy of epigenetic inference of heavy drinkers *vs.* light and non-drinkers using different marker sets.

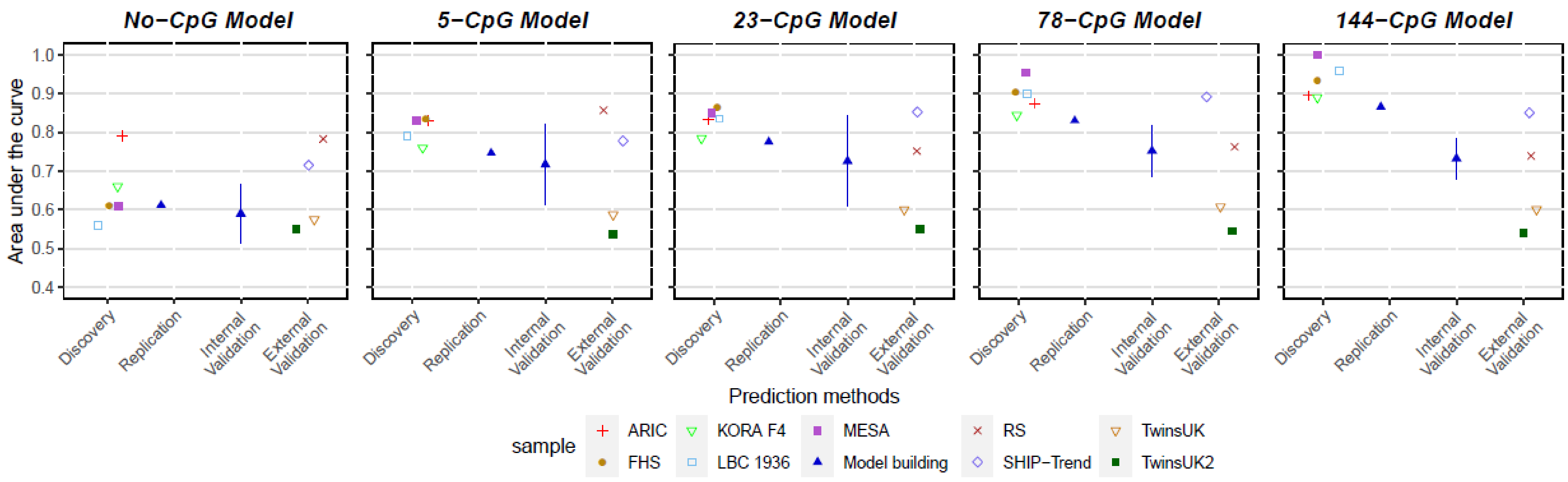

Figure S9. Accuracy of epigenetic inference of heavy drinkers *vs.* at-risk drinkers using different marker sets.

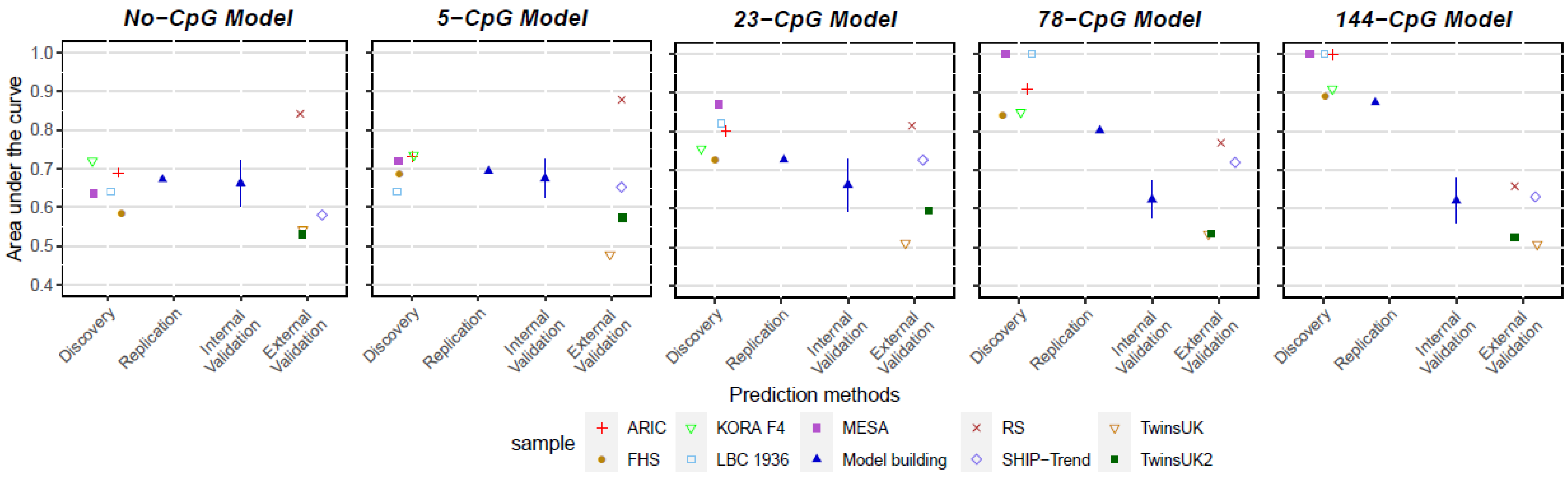

Figure S10. Accuracy of epigenetic inference of at-risk drinkers *vs.* non-drinkers using different marker sets.

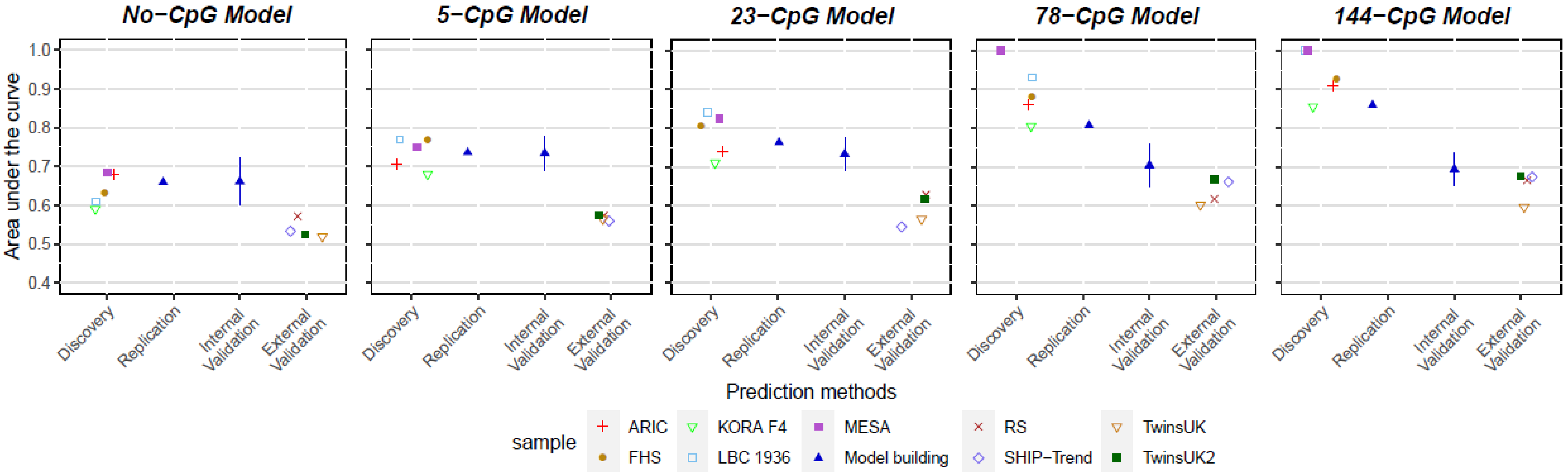

Figure S11. Accuracy of epigenetic inference of at-risk drinkers *vs.* light drinkers using different marker sets.

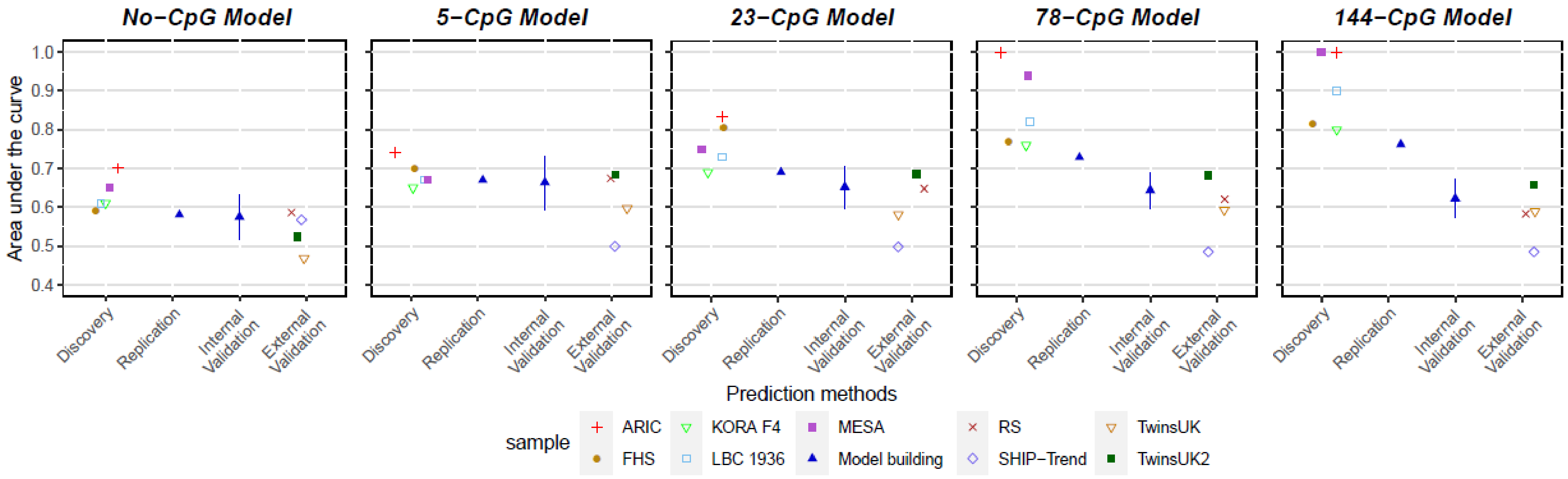

Figure S12. Accuracy of epigenetic inference of light drinkers *vs.* non-drinkers using different marker sets.

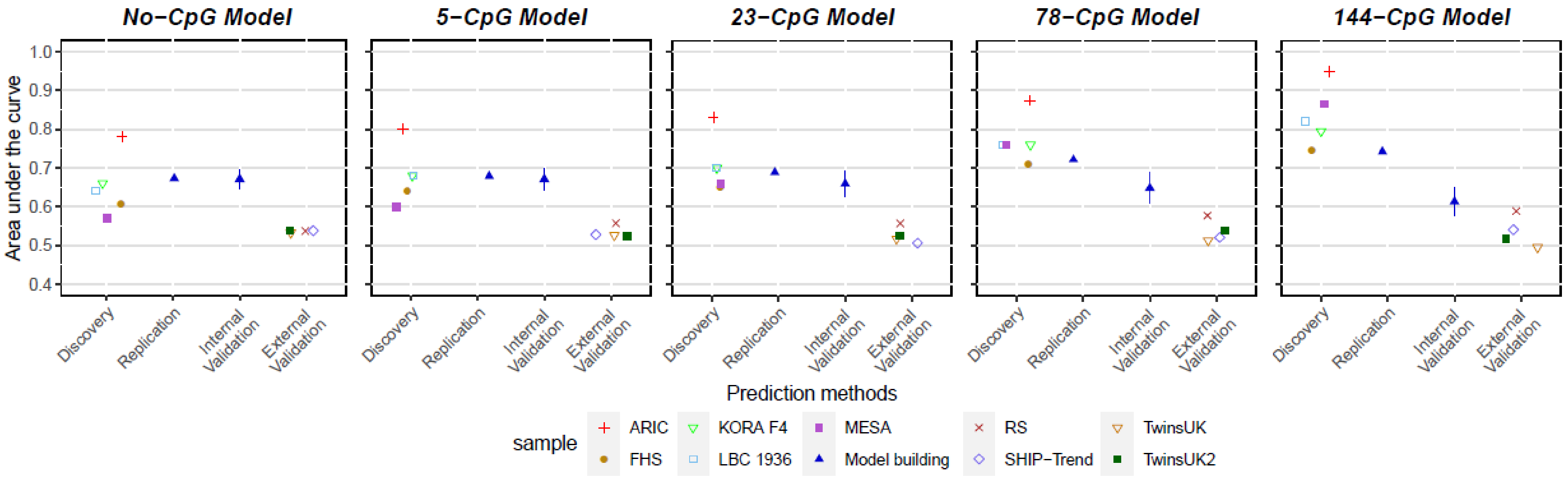

Figure S13. Accuracy of epigenetic inference of heavy and at-risk drinkers *vs.* light and non-drinkers using different marker sets.

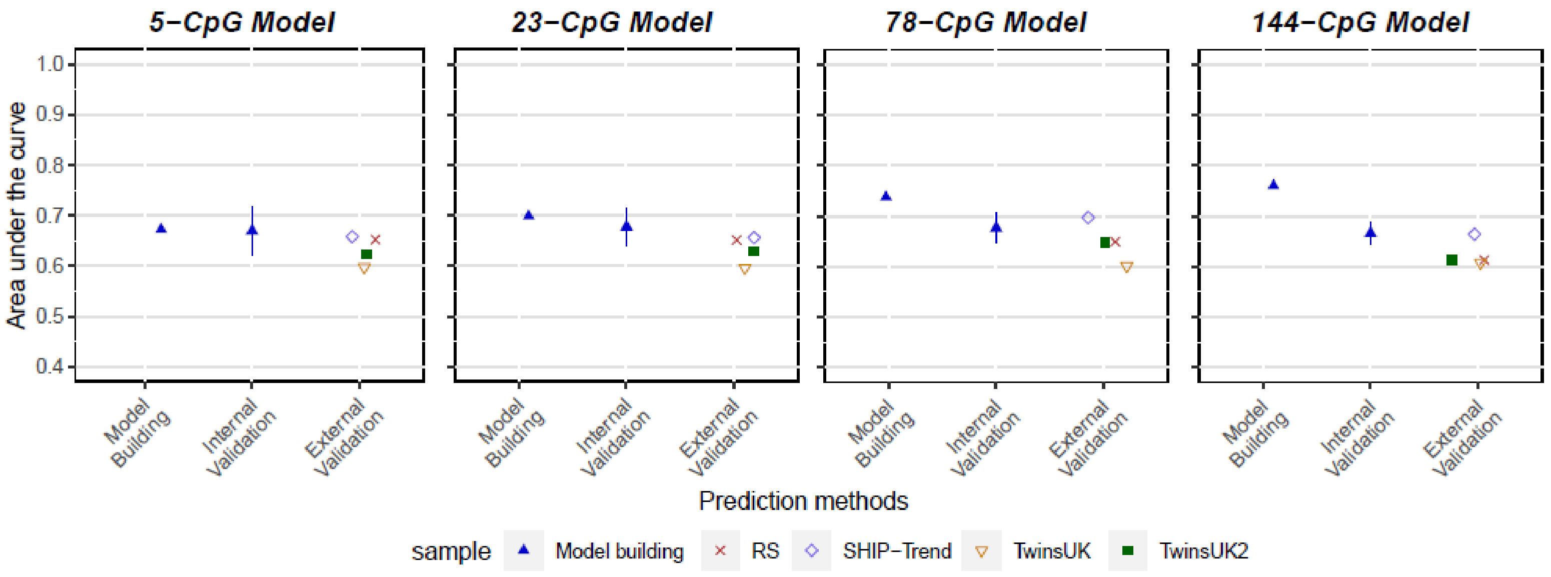

Figure S14. Accuracy of epigenetic inference of heavy, at-risk and light drinkers *vs.* non-drinkers using different marker sets.

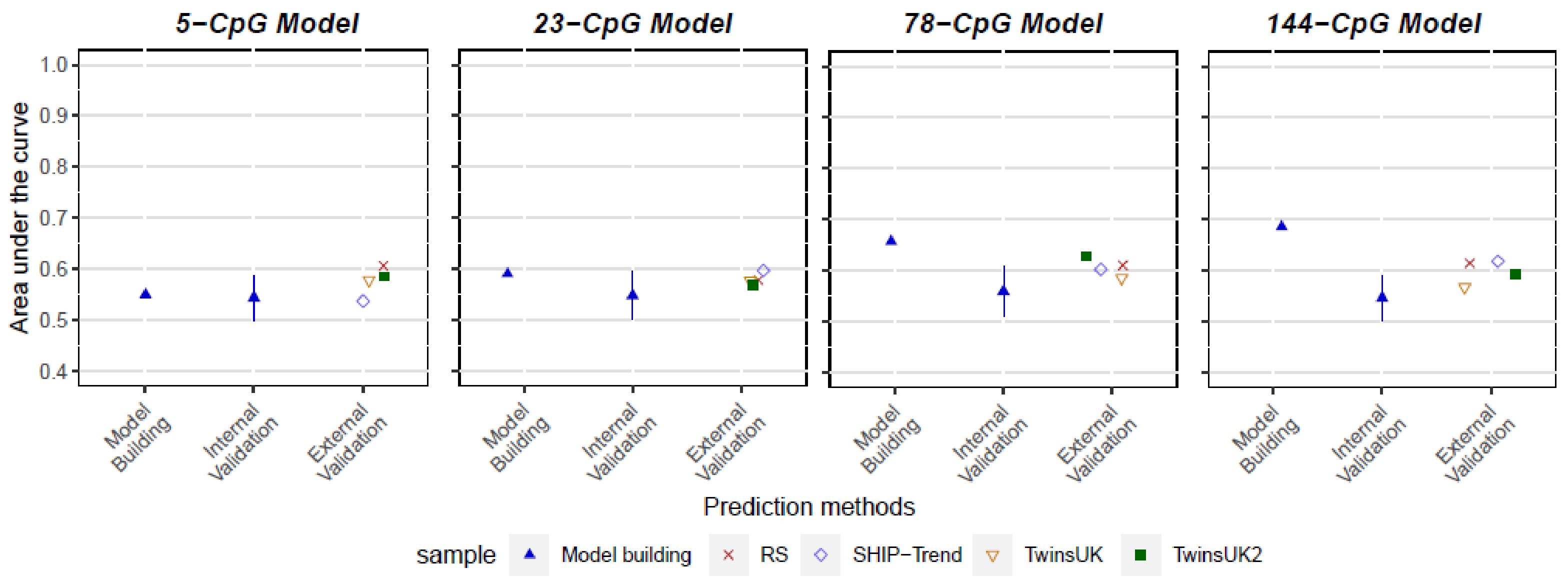

Reference

1. Liu C, Marioni RE, Hedman AK, Pfeiffer L, Tsai PC, Reynolds LM, et al. A DNA methylation biomarker of alcohol consumption. Mol Psychiatry. 2018;23(2):422-33.
2. Ikram MA, Brusselle G, Ghanbari M, Goedegebure A, Ikram MK, Kavousi M, et al. Objectives, design and main findings until 2020 from the Rotterdam Study. Eur J Epidemiol. 2020;35(5):483-517.
3. van Greevenbroek MM, Jacobs M, van der Kallen CJ, Vermeulen VM, Jansen EH, Schalkwijk CG, et al. The cross-sectional association between insulin resistance and circulating complement C3 is partly explained by plasma alanine aminotransferase, independent of central obesity and general inflammation (the CODAM study). Eur J Clin Invest. 2011;41(4):372-9.
4. Willemsen G, Vink JM, Abdellaoui A, den Braber A, van Beek JH, Draisma HH, et al. The Adult Netherlands Twin Register: twenty-five years of survey and biological data collection. Twin Res Hum Genet. 2013;16(1):271-81.
5. Schoenmaker M, de Craen AJ, de Meijer PH, Beekman M, Blauw GJ, Slagboom PE, et al. Evidence of genetic enrichment for exceptional survival using a family approach: the Leiden Longevity Study. Eur J Hum Genet. 2006;14(1):79-84.
6. Huisman MH, de Jong SW, van Doormaal PT, Weinreich SS, Schelhaas HJ, van der Kooi AJ, et al. Population based epidemiology of amyotrophic lateral sclerosis using capture-recapture methodology. J Neurol Neurosurg Psychiatry. 2011;82(10):1165-70.
7. Bonder MJ, Luijk R, Zhernakova DV, Moed M, Deelen P, Vermaat M, et al. Disease variants alter transcription factor levels and methylation of their binding sites. Nat Genet. 2017;49(1):131-8.
8. Holle R, Happich M, Lowel H, Wichmann HE, Group MKS. KORA--a research platform for population based health research. Gesundheitswesen. 2005;67 Suppl 1:S19-25.
